# Electron Microscopy Techniques for Investigating Structure and Composition of Hair-Cell Stereociliary Bundles

**DOI:** 10.3389/fcell.2021.744248

**Published:** 2021-10-22

**Authors:** Maryna V. Ivanchenko, Artur A. Indzhykulian, David P. Corey

**Affiliations:** ^1^Department of Neurobiology, Harvard Medical School, Boston, MA, United States; ^2^Department of Otolaryngology, Massachusetts Eye and Ear, Harvard Medical School, Boston, MA, United States

**Keywords:** hair cell, cochlea, stereocilia, electron microscopy, SEM, TEM, FIB-SEM, immunogold

## Abstract

Hair cells—the sensory cells of the vertebrate inner ear—bear at their apical surfaces a bundle of actin-filled protrusions called stereocilia, which mediate the cells’ mechanosensitivity. Hereditary deafness is often associated with morphological disorganization of stereocilia bundles, with the absence or mislocalization within stereocilia of specific proteins. Thus, stereocilia bundles are closely examined to understand most animal models of hereditary hearing loss. Because stereocilia have a diameter less than a wavelength of light, light microscopy is not adequate to reveal subtle changes in morphology or protein localization. Instead, electron microscopy (EM) has proven essential for understanding stereocilia bundle development, maintenance, normal function, and dysfunction in disease. Here we review a set of EM imaging techniques commonly used to study stereocilia, including optimal sample preparation and best imaging practices. These include conventional and immunogold transmission electron microscopy (TEM) and scanning electron microscopy (SEM), as well as focused-ion-beam scanning electron microscopy (FIB-SEM), which enables 3-D serial reconstruction of resin-embedded biological structures at a resolution of a few nanometers. Parameters for optimal sample preparation, fixation, immunogold labeling, metal coating and imaging are discussed. Special attention is given to protein localization in stereocilia using immunogold labeling. Finally, we describe the advantages and limitations of these EM techniques and their suitability for different types of studies.

## Introduction

Hair cells of the vertebrate inner ear bear at their apical surfaces a bundle of 30–300 actin-filled protrusions called stereocilia. Stereocilia mediate the hair cells’ mechanosensitivity, both by their precise organization in rows of increasing height and with the force-gated ion channel complexes located at their tips (Assad et al., 1991; Gillespie and Walker, 2001; Geleoc and Holt, 2003; Beurg et al., 2009; Zhao and Muller, 2015; Fettiplace, 2017; Velez-Ortega and Frolenkov, 2019). Stereocilia are cross-linked by a variety of transient or permanent links which contribute to the integrity of the bundles—shaping the bundle during the development and keeping the proper stereocilia arrangement thereafter (Pickles et al., 1984; Goodyear et al., 2005).

Mutations in more than 120 genes have been shown to cause deafness in humans, and many more genes remain to be discovered (Van Camp, 2021)^1^. Hereditary deafness is often associated with morphological disorganization of stereocilia bundles, as either a primary or secondary consequence of the gene defect, and is often associated with an absence or mislocalization of specific proteins within stereocilia (Dror and Avraham, 2010; Richardson et al., 2011). Thus, stereocilia bundles are closely examined to understand most animal models of hereditary hearing loss. Because stereocilia have a diameter less than a wavelength of light, light microscopy is not always adequate to reveal subtle changes in morphology or protein localization. Transmitted or confocal light microscopy may hint at stereocilia defects, but electron microscopy is usually needed for full characterization.

For many years, electron microscopy (EM) techniques such as transmission electron microscopy (TEM; Furness and Hackney, 1985; Kachar et al., 2000; Goodyear et al., 2005; Mogensen et al., 2007; Grillet et al., 2009; Karavitaki and Corey, 2010; Mahendrasingam et al., 2017) and scanning electron microscopy (SEM; Furness and Hackney, 1985; Kachar et al., 2000; Mogensen et al., 2007; Grillet et al., 2009; Verpy et al., 2011; Indzhykulian et al., 2013; Fang et al., 2015; Parker et al., 2016; Mahendrasingam et al., 2017; Velez-Ortega et al., 2017; Ivanchenko et al., 2020b, 2021) have been used to study hair-cell bundles, and EM has proven to be essential for understanding stereocilia bundle development, maintenance, normal function, and dysfunction in disease. Immunogold electron microscopy techniques have been increasingly used in hearing research, providing excellent insight into structure-function relationships and protein distribution within the cell (Hasson et al., 1997; Garcia et al., 1998; Siemens et al., 2004; Furness et al., 2005; Michel et al., 2005; Goodyear et al., 2010; Verpy et al., 2011; Chen et al., 2012; Indzhykulian et al., 2013; Fang et al., 2015; Mahendrasingam et al., 2017; Wu et al., 2019; Han et al., 2020; Ivanchenko et al., 2020a).

These methods differ in their underlying physics and sample preparation, and each has advantages for certain experiments. TEM is a method in which a focused beam of electrons passes through an ultrathin 60–100 nm or semithin 250–500 nm section (Furness et al., 2008) specimen placed in an image plane of the electron optics (Bozzolla and Russell, 1998). The sample has been fixed and stained with heavy elements (e.g., osmium tetroxide, potassium ferrocyanide, uranyl acetate, lead citrate) which attach to certain cellular structures, thus enhancing the electron density of some parts of the cell more than others (Glauert and Lewis, 1998). The electrons passing through the samples are scattered more by the denser regions, resulting in dark regions on the projected image. TEM is useful in the analysis of almost all cellular components, including the cytoskeleton, membrane systems, organelles, etc. With new digital TEM cameras, 0.2–0.5 nm resolution in the X-Y plane can be obtained when working with biological samples.

The use of antibodies that are conjugated to 5–25 nm colloidal gold beads, used to localize proteins in cells, has been a major addition to EM (Jones, 2016). As an example, SEM enabled the initial discovery of the tip link (Osborne et al., 1984; Assad et al., 1991; Siemens et al., 2004), which pulls on the force-gated channel complex, but immunogold labeling confirmed its molecular composition (Ahmed et al., 2006; Kazmierczak et al., 2007; Sakaguchi et al., 2009; Goodyear et al., 2010; Indzhykulian et al., 2013). Many different protocols for immunogold TEM (IG-TEM) have been developed. The most commonly used techniques are either immunogold labeling before the sample is embedded in resin (pre-embedding) or immunogold labeling after embedding in resin (post-embedding) (Jones, 2016). In post-embedding immunolabeling, unfixed tissue is high-pressure frozen or fixed with formaldehyde, with or without a small amount of glutaraldehyde, then embedded in acrylic resin, e.g., Lowicryl (low-temperature embedding) or the hydrophilic LR-White resin (room temperature embedding and polymerization). Then blocks are sectioned and ultrathin sections are immunolabeled. The advantage of this method is that it can label proteins regardless of their location (intracellular or extracellular) and offers best ultrastructural preservation when adequate fixation is applied (Boykins et al., 2016; Jones, 2016). Also, a single block of tissue (e.g., a whole mouse cochlea) prepared for TEM yields thousands of sections to label with different antibodies, or alternatively can be used in serial section immunolabeling (Furness et al., 2009). Careful orientation of the sections allows visualization of several turns in cross-section and thus comparisons along the cochlear gradient within the same section. Estimates using calibrated immunogold with post-embedding immunogold labeling have enabled absolute protein densities to be calculated, for instance for PMCA2 in the stereociliary membrane (Chen et al., 2012) and for different calcium buffers in stereocilia compartments vs. the cell cytoplasm (Hackney et al., 2005). In several studies, relative protein concentrations have been compared between cells. Finally, double (or even triple) labeling can be achieved relatively easily with post-embedding immunogold using different-sized gold particles (Mahendrasingam et al., 2011). However, the limitation is that the antibodies have difficulty penetrating into the resin, and as a result, only the antigens that are exposed on the surface of the section can be immunolabeled.

In pre-embedding immunolabeling, the sample is fixed with a “gentle” fixative like formaldehyde, then immunolabeled. The cells are then post-fixed with glutaraldehyde and processed for SEM (Goodyear et al., 2010; Indzhykulian et al., 2013; Wu et al., 2019) or embedded in epoxy resin, blocks are sectioned, and ultrathin sections are imaged with TEM (Jones, 2016; Wu et al., 2019). Double labeling has been achieved with pre-embedding immunolabeling for extracellular epitopes (Goodyear et al., 2010). In contrast to post-embedding, pre-embedding immunolabeling offers better epitope preservation and better antibody binding, and thus a stronger signal. The limitation is that it works best for extracellular proteins where the epitopes are accessible to antibodies, while some intracellular epitopes can be labeled following permeabilization. The labeling procedures require extended incubations in buffers which leads to reduced ultrastructural preservation since formaldehyde is a weaker fixative than glutaraldehyde. Also, pre-embedding allows a one-off labeling run and is thus more costly in terms of animals, material and time. A protocol in this paper describes a pre-embedding procedure for extracellular proteins that gives reliable results with almost perfect preservation of the ultrastructure.

In contrast to TEM, which allows to image intracellular compartments and organelles on thin sections, SEM is used to visualize surfaces and identify proteins on surfaces (Fischer et al., 2012). With SEM, an electron beam focused to a point scans the surface of a sample coated with a thin layer of conductive material, like platinum, palladium, or gold. In one imaging mode, the incident electrons excite metal atoms of the coating which emit secondary electrons (SE) that are collected by a detector. In another, incident electrons are scattered back, often by heavy metal beads; backscattered electrons (BSE) collected by a different detector. Following a raster scan, an image is created. The result is a two-dimensional (2-D) image of the sample surface; because of shading created from the topology of the specimen’s surface and consequently by the angle of incidence of the beam, it appears three-dimensional (3-D). However, the metal sputter coating generates artifactual structures on the surfaces of stereocilia giving some increase in dimensions (e.g., of tip links) that become visible with higher resolution imaging. The use of the OTOTO technique (Heywood and Resnick, 1981; Furness and Hackney, 1985) (osmium tetroxide, followed by thiocarbohydrazide, osmium tetroxide, thiocarbohydrazide, osmium tetroxide) is an advantage in many cases. Thiocarbohydrazide serves as the cross-linking agent and allows more osmium to be taken up by the sample, thus eliminates the need for metal coating. The most common mode in SEM is SE detection, in which the electrons are emitted from the surface or the near-surface areas of the sample, and an image of the surface is created. In contrast, the BSE detection mode provides information about elemental composition of the scattering surface, with high sensitivity to differences in atomic number. Heavier atoms (with a higher atomic number) scatter electrons more strongly than lighter atoms and therefore produce a stronger signal, which appears brighter in the image. Using BSE, imaging can be achieved with nanometer resolution for detection and localization of proteins or other biological molecules immunolabeled with colloidal gold beads on the surface of the cell membrane (IG-SEM).

Serial EM techniques have added a third (Z) dimension, for a more complete representation of biological structure. These methods have become more accessible for use in hearing research, enabling a new body of work using 3-D EM approaches (Vranceanu et al., 2012; Bullen et al., 2015; Katsuno et al., 2019; Wu et al., 2019; Hadi et al., 2020; Ivanchenko et al., 2020a; Hua et al., 2021; Lu et al., 2021; Payne et al., 2021; Wood et al., 2021). The most commonly used 3-D EM techniques are serial section TEM (Harris et al., 2006), focused-ion-beam scanning electron microscopy (FIB-SEM; Kizilyaprak et al., 2014), and serial block-face SEM (SBF-SEM; Denk and Horstmann, 2004). Powerful but less commonly used is electron tomography (Auer et al., 2008).

Serial section TEM was the pioneer among the 3-D EM techniques, used first in the 1950s (Gay and Anderson, 1954). Consecutive thin sections, prepared with a routine ultramicrotome and collected on formvar coated slot or hole grids then are imaged with a conventional TEM and used for 3-D analyses (Miranda et al., 2015). Although technically demanding, it is a very data-rich method. The serial sections, if stored properly and imaged carefully, can be re-investigated many times for different aspects of a structure or to retake the sequence. Some techniques utilize tape-collecting ultramicrotomes to collect serial sections to be imaged with SEM (Goodyear et al., 2010; Baena et al., 2019) or TEM (Bock et al., 2011; Yin et al., 2020), in some cases automatically feeding the tape with EM sections into the microscope (Phelps et al., 2021). Another technique, serial block-face scanning electron microscopy (SBF-SEM), sections the specimen via an ultramicrotome built inside the SEM chamber. Each newly exposed face is imaged, followed by cutting and imaging a new face in an automated fashion (Denk and Horstmann, 2004). Finally, electron tomography consists of acquiring a tilt series of projected images in the electron microscope, followed by a number of image processing and digital reconstruction steps that generate a 3-D volume. It is especially useful for studies at the single protein level, or to avoid serial sectioning (Miranda et al., 2015). All these techniques enable 3-D serial reconstruction of resin-embedded biological structures at a resolution of a few nanometers in the X-Y plane, but vary in their Z step size and the optimal imaging area.

The FIB-SEM instrument is a combination of an SEM, usually equipped with a field-emission source, with a focused ion beam (FIB) of gallium ions that etch a sample embedded in epoxy resin. In FIB-SEM, a 5–20 nm slice is removed with the ion beam, and the newly exposed surface is imaged by the SEM backscatter detector. The process, called “slice and view,” is repeated hundreds of times until a volume of cubic micrometers is imaged. The combination of imaging with an electron beam and slicing with the FIB in a dual-beam electron microscope opened new possibilities for serial imaging and 3-D reconstruction with electron microscopic resolution.

Although conventional FIB-SEM can be used to visualize a volume containing several cells in three dimensions with high resolution, it still does not provide information about protein localization. The use of immunogold labeled samples has overcome this limitation. Immunogold FIB-SEM (IG-FIB-SEM) can correlate biomolecular and structural information with high spatial resolution and in large volumes (Gopal et al., 2019; Wu et al., 2019; Ivanchenko et al., 2020a).

These methods vary in their resolution, speed and ability to image different tissues and proteins. Deciding which EM technique is best is sometimes not straightforward, and a combination of EM methods may eventually be chosen to address a single question. Here we describe the specific methods and imaging protocols for each technique. Parameters for optimal sample preparation, fixation, immunogold labeling, metal coating, and imaging are discussed. Special attention is given to protein localization in stereocilia using immunogold labeling. Finally, we describe the advantages and limitations of these EM techniques and their suitability for different types of studies.

## Materials and Methods

### General Instructions

1. Most chemicals used for EM are extremely hazardous, for instance carcinogenic or flammable, so general lab safety rules must be followed. Wear a lab coat, gloves, and protective eyewear whenever working with chemicals. Solutions should be prepared and used in a fume hood. Hazardous material waste must be collected and disposed of in accordance with Hazardous Waste Management guidelines.
2. Because EM sample preparation is a multi-step process, each step affects the final result, extreme fidelity to the protocol is essential. A mistake in any step can compromise the result.
3. An essential factor in EM is the quality of the water used for all the buffers and the solutions. Ultrapure water is recommended.
4. It is important to use a freshly prepared fixative at the same temperature as the tissue itself. Furthermore, it must be applied without delay. The best results are obtained if the fixative reacts with the sample within a few seconds after the removal from the cochlea. The volume of the fixative should exceed the volume of the sample by at least 20 times.
5. It is important to cut samples into pieces no larger than 1–2 mm. This allows rapid penetration of the chemicals, thereby ensuring ultrastructural integrity.
6. Stereocilia are very delicate structures and the integrity of hair cell bundles can be easily disrupted during the washing steps. Never touch the organ of Corti with the pipet tip, and exchange the solutions at a low speed.
7. Large specimens (such as an entire cochlea) must be kept in the dehydration solution for a longer period of time to enable complete removal of water from the specimen. They also require prolonged exposure to osmium tetroxide and extended infiltration in the embedding resin to allow these substances to fully penetrate the sample (Spoendlin and Brun, 1974; Bohne and Harding, 1993).
8. Fixation modifies the structure of antigens, often making them undetectable by specific antibodies. The time and type of fixation should be considered, especially for antibodies that are known to lose their specificity following antigen fixation. Also, antibodies can be made to glutaraldehyde fixed antigens.
9. If possible, previously published antibodies that have been successfully used for immunofluorescence labeling should be considered as a first choice. If using a newly produced antibody, validation experiments should be performed to determine their specificity, selectivity, and reproducibility.

### Tissue Preparation

Animal handling, breeding, and all procedures were performed in compliance with NIH Ethics guidelines and with a protocol approved by the Animal Care Committee of Harvard Medical School (HMS) and Massachusetts Eye and Ear. Mice were housed and bred at the HMS or the Massachusetts Eye and Ear animal facilities.

Neonatal mice (P1–P6) are anesthetized with cryoanesthesia. After the pup no longer responds to painful stimuli it is decapitated at the base of the head (foramen magnum). To access the cochlea, the cranium is opened along the sagittal suture and the caudal forebrain is cut off. Cochleas are isolated from the temporal bones in room temperature Leibovitz’s L-15 medium without phenol red (GIBCO, 21083027). The bony labyrinth from each cochlea is removed by careful separation starting from the apex toward the base, using fine forceps. The organ of Corti is unwound from the modiolus. The spiral ligament is then removed from the organ of Corti using fine forceps. Organs then are immediately transferred to the fixative solution using a glass serological pipette with enlarged opening, or a transfer spoon, to prevent the tissue from passing through the water-air interface.

Adult mice (P30 and P90) are anesthetized with isoflurane. After they no longer respond to painful stimuli, they are euthanized by cervical dislocation and decapitated. Cochleas are extracted into a plastic dish filled with a room temperature Leibovitz’s L-15 solution. The stapes is removed from the oval window and the round window membrane punctured with fine forceps, to allow fixative to flow through the cochlea. Under a stereomicroscope, a small hole is made in the apex of the cochlea using a 27G needle connected to a 1 ml syringe. Cochleas then are immediately transferred to the fixative solution.

We have found that temperature is not critical and processing on ice is not necessary. All dissection and fixation steps are performed at room temperature.

### Preparation of Solutions

#### Solutions for Cochlear Dissection

Leibovitz’s L-15 medium, no phenol red (GIBCO, 21083027) is often used. The medium contains amino acids, vitamins, inorganic salts, and other components to maintain cell health and survival in environments without CO_2_ equilibration. It includes 1.2 mM Ca^2+^, essential to preserve the tip links.

#### Solutions for Rinsing

(a) Sodium cacodylate buffer, 0.1 M, pH 7.4, 100 ml is prepared by mixing 50 ml of commercial sodium cacodylate buffer, 0.2 M, pH 7.4 [Electron Microscopy Sciences (EMS), 11652] with 50 ml distilled H_2_O (dH_2_O). This solution can be prepared in advance and stored at 0–5°C. It can be used within 2–3 months. *The sodium cacodylate buffer contains arsenic, which is poisonous and carcinogenic.*
(b) Hank’s balanced salt solution (HBSS), with calcium and magnesium, no phenol red, 500 ml (GIBCO, 14025092).
(c) HBSS, with no calcium or magnesium, no phenol red, 500 ml (GIBCO, 14175095).

#### Solutions for Fixation

Always use electron microscopy or analytical grade reagents.

(a) 2.5% glutaraldehyde in 0.1 M cacodylate buffer (pH 7.2) supplemented with 2 mM CaCl_2_ is prepared by adding 10 μl of 2 M CaCl_2_ stock solution to 10 ml of 2.5% glutaraldehyde in 0.1 M sodium cacodylate buffer, pH 7.4 (EMS, 15960). To make 100 ml of 2 M CaCl_2_, dissolve 29.4 g of calcium chloride dihydrate (EMS, 12340) in 100 ml of dH_2_O.
(b) 4% formaldehyde in HBSS with calcium and magnesium, no phenol red (pH 7.2) is prepared by mixing 10 ml of 16% formaldehyde aqueous solution (EMS, 15700) in 30 ml of HBSS with calcium and magnesium, no phenol red (pH 7.2) (GIBCO, 14025092).
(c) 4% formaldehyde/1% glutaraldehyde fixative in 0.1 M sodium cacodylate buffer (100 ml) can be prepared by mixing 25 ml of 16% formaldehyde aqueous solution (EMS, 15700) with 2 ml of 50% glutaraldehyde aqueous solution (EMS, 16300), 50 ml of sodium cacodylate buffer, 0.2 M, pH 7.4 (EMS, 11652), and 23 ml dH_2_O.
(d) 2.5% glutaraldehyde in 0.1 M cacodylate buffer (pH 7.2) containing 1% (or 5%) tannic acid and supplemented with 2 mM CaCl_2_ is prepared by dissolving 0.1 g (or 0.5 g) of tannic acid, A.C.S. (EMS, 21710) with 10 ml 2.5% glutaraldehyde in 0.1 M cacodylate buffer (pH 7.2) supplemented with 2 mM CaCl_2_ (see a).

#### Solutions for Staining

(a) 2% aqueous osmium tetroxide is prepared by diluting 5 ml of 4% aqueous osmium tetroxide (EMS, 26604-01) in 5 ml of dH_2_O. *Osmium tetroxide vapors can cause burns or severe irritation of the skin, respiratory tract, and eyes, and can cause long-term health effects.*
(b) 1% osmium tetroxide/1.5% potassium ferrocyanide in 0.1 M cacodylate buffer, (pH 7.2) is prepared right before use by mixing a solution containing 3% potassium ferrocyanide in 0.2 M cacodylate buffer with 4 mM CaCl_2_ with an equal volume of 2% aqueous osmium tetroxide.
(c) 3% stock potassium ferrocyanide in 0.2 M cacodylate buffer with 4 mM CaCl_2_ is prepared by dissolving 0.3 g of potassium ferrocyanide trihydrate (EMS, 26604-01) in 10 ml of 0.2 M sodium cacodylate buffer (EMS, 11652) and supplementing with 20 μl of 2 M CaCl_2_ stock.
(d) 1% aqueous tannic acid is prepared right before use by dissolving 0.1 g of tannic acid, A.C.S. (EMS, 21710) in 10 ml dH_2_O.

#### Solutions for Dehydration

For the dehydration step, about 50 ml of the following solutions are needed. These solutions can be prepared in advance and stored in tightly closed glass bottles at room temperature for a long time. *Ethanol and propylene oxide are flammable; propylene oxide is carcinogenic.*

(a) 25, 50, 70, 80, and 95% ethanol in dH_2_O, 50 ml each.
(b) Ethanol, absolute (200 proof, molecular biology grade).
(c) Propylene oxide (EMS, 20401).
(d) Propylene oxide/ethanol (1:1).

#### Embedding Resins

(a) Araldite 502/Embed 812 embedding medium without the accelerator (EMS, 13940) can be prepared by pouring measured volumes of Embed-812 (12.5 ml); DDSA (27.5 ml), Araldite 502 (7.5 ml) into a graduated 50 ml polypropylene tube with a tight cap. The best way to accomplish this is to warm components for 15–20 min in the oven (60°C) to reduce their viscosity prior to measuring and mixing the resin. After pouring the warmed resin into the 50 ml polypropylene tube, tighten the cap and keep it on the tube rocker for 10–15 min. For larger batches, the components should be increased proportionally. Unused embedding mix can be stored for up to 6 months at 4–8°C in a tightly closed container. In order to prevent rapid polymerization of the resin during storage, ensure that stored resin mixture does not include an accelerator. However, it is preferable to use a freshly prepared embedding medium. If using a previously stored mixture, it has to be warmed before adding the accelerator. Mix components thoroughly, avoiding bubbles, as they can interfere with the tissue embedding. Plan enough time between mixing the resin and the embedding step for the bubbles to disappear.
(b) 3:1 solution of propylene oxide/Araldite 502/Embed 812 embedding medium can be prepared by mixing 30 ml of propylene oxide (EMS, 20401) with 10 ml of Araldite 502/Embed 812 embedding medium without the accelerator.
(c) 1:1 solution of propylene oxide: Araldite 502/Embed 812 embedding medium can be prepared by mixing 20 ml of propylene oxide (EMS, 20401) with 20 ml of Araldite 502/Embed 812 embedding medium without the accelerator.
(d) 1:3 solution of propylene oxide: Araldite 502/Embed 812 embedding medium can be prepared by mixing 10 ml of propylene oxide (EMS, 20401) with 30 ml of Araldite 502/Embed 812 embedding medium without the accelerator.
(e) Araldite 502/Embed 812 embedding medium with accelerator can be prepared by adding 100 μl of DMP-30 to 5 ml Araldite 502/Embed 812 embedding medium. It has to be prepared just before use to avoid rapid polymerization. The components should be mixed thoroughly using a tube rocker for 10–15 min; avoid creating bubbles.

*Epoxy resin monomers can cause severe contact dermatitis and are carcinogenic. Propylene oxide can carry resins into the skin even through gloves.*

Although our choice of resin is Araldite 502/Embed 812 (low viscosity epoxy resin), other resins such as Epon 812 (Katsuno et al., 2019) (low viscosity epoxy resin), Spurr’s resin (Hua et al., 2021; Wang et al., 2021) (less viscous than Epon mixtures), Lowicryl HM-20 (Velez-Ortega et al., 2017) (formulated to provide low viscosity at low temperatures), LR-White (Furness et al., 2008; Mahendrasingam et al., 2011) (hydrophilic acrylic resin), and Durcupan (Dow et al., 2018) (water-miscible epoxy resin) have been successfully used to study hair cell stereocilia bundles.

#### Solutions for Immunogold Labeling

A blocking reagent is used to reduce background from non-specific, conserved sequence and/or Fc receptor binding sites. Normal serum (10%) is diluted in HBSS, with no calcium or magnesium, no phenol red (GIBCO, 14175095). The best results are obtained using the normal serum from the same host as the secondary antibody.

(a) 10% normal goat (or donkey) serum is prepared by diluting 1 ml of stock normal goat (or donkey) serum in 9 ml of HBSS with no calcium or magnesium, no phenol red (GIBCO, 14175095). It has to be prepared freshly and stored at 4°C for use within 48 h. An alternative solution for HBSS is Tris-buffered saline (TBS).
(b) Some vendors provide the serum as a dry, lyophilized powder. In that case, the stock of normal goat (or donkey) serum is prepared by dissolving the serum (Jackson ImmunoResearch, 005-000-121) in 10 ml of dH_2_O. If the solution is not clear, it is advisable to use centrifugation. The stock product can be aliquoted and stored frozen at −20°C for 1 year; avoid repeated freeze-thaw cycles.
(c) The desired concentrations of solutions for the primary and secondary antibody are prepared in blocking solutions (10% normal serum, see above). The antibody solution must be prepared freshly before use and should be kept cold, on an ice box.
(d) In this work, we used the following antibodies:

- Rabbit anti-PKHD1L1 primary antibody (NovusBio, NBP2-13765), 1:200 dilution in blocking solution.
- Rabbit anti-PCDH15 primary antibody (DC 811), 1:200 dilution in blocking solution. This antibody has been custom made using the same epitope as the previously reported and well-characterized PB811 (Kazmierczak et al., 2007).
- Rabbit anti-STRC primary antibody raised against amino acids 970–985 of the mouse stereocilin protein (Han et al., 2020) 0.12 nm Colloidal Gold AffiniPure Goat Anti-Rabbit IgG (EM Grade) secondary antibody (Jackson ImmunoResearch, 111-205-144), 1:30 dilution in blocking solution.
- Gold Conjugate EM Goat F(ab’)2 anti-rabbit IgG: 10 nm gold secondary antibody (BB International #14216), 1:30 dilution in blocking solution.

#### Post-staining Solutions

(a) 1% methylene blue/1% sodium borate solution can be prepared by first dissolving 2 g of methylene blue crystals (EMS, 22050) in 100 ml dH_2_O. The sodium borate solution can then be prepared by dissolving 2 g of sodium borate (EMS, 21130) in 100 ml dH_2_O. Filter the solutions through a 0.22 μm syringe filter. The two solutions should be mixed 1:1 and stored in a brown bottle.
(b) 5% methanolic uranyl acetate (EMS, 22400) is prepared by dissolving 5 g of uranyl acetate in 48 ml of near-boiling CO_2_-free double-distilled water. Once the uranyl acetate crystals are fully dissolved, the solution is filtered through a 0.22 μm syringe filter and additionally diluted with 48 ml of acetone-free methanol. The advantage of a methanolic uranyl acetate is that it penetrates more easily into plastic sections giving a higher contrast. Because uranyl acetate is light sensitive, it should be stored in a 100 ml glass brown bottle, or the container should be wrapped with aluminum foil to exclude light, and capped tightly. This stock solution can be stored at 4°C for several months.
(c) Lead citrate is prepared as described by Reynolds (1963) by dissolving 1.33 g of lead nitrate (EMS, 17900) and 1.76 g of sodium citrate (EMS, 21140) in 30 ml of freshly boiled and cooled, deionized, or distilled water in a 50 ml volumetric flask. The milky suspension is shaken vigorously for 1 min, followed by continued intermittent shaking to ensure complete conversion of lead nitrate to lead citrate. After 30 min, 8.0 ml of carbonate-free, 1 N NaOH solution is added, making the solution clear. Carbonate-free, 1 N NaOH solution purchased from a reliable vendor is preferable to freshly prepared solutions made in the laboratory. Dilute the resulting solution to 50 ml with freshly boiled deionized or distilled water. The Reynold’s solution may be stored in a glass-stoppered bottle for up to 6 months. The pH of the final solution should be about 12.0. In addition, an improved method for storing stains has been described (Reynolds, 1963). Before use, pass the solution through a microfilter.

An alternative protocol for immediate use is to dissolve lead citrate compound (between 0.1 and 0.4 mg in 10 ml ultrapure water) at high pH by adding 4 N NaOH.

### Step-by-Step Procedures Used in This Study

#### Protocol for Conventional Scanning Electron Microscopy

##### Fixation

P1–P3 cochlear explants are fixed immediately after dissection, with 2.5% glutaraldehyde in 0.1 M cacodylate buffer (pH 7.2) supplemented with 2 mM CaCl_2_, for 2 h at room temperature. The samples are rinsed three times in sodium cacodylate buffer, 0.1 M, pH 7.4 for 10 min, and then briefly once in distilled water.

P4–P7 explants are prefixed immediately after dissection with 4% formaldehyde in HBSS with calcium and magnesium, no phenol red (pH 7.2) for 10–15 min, and then transferred to HBSS with calcium and magnesium, no phenol red (pH 7.2). Under a dissecting microscope, the tectorial membrane overlaying the hair cells is pulled away using fine forceps to expose the sensory epithelium. The organ of Corti is postfixed with 2.5% glutaraldehyde in 0.1 M cacodylate buffer (pH 7.2) supplemented with 2 mM CaCl_2_ for 2 h at room temperature. Samples are rinsed three times in sodium cacodylate buffer, 0.1 M, pH 7.4 for 10 min, and then briefly once in distilled water.

Adult cochleas are prefixed immediately after dissection by immersion in 1% glutaraldehyde/4% formaldehyde in 0.1 M cacodylate buffer (pH 7.2) supplemented with 2 mM CaCl_2_, for 1 h at room temperature. Additionally, samples are gently and slowly perfused with 1% glutaraldehyde/4% formaldehyde solution via the round and oval windows until the solution is washed out of the small hole at the apex. After the prefixation step, the samples are fixed with 2.5% glutaraldehyde in 0.1 M cacodylate buffer (pH 7.2) supplemented with 2 mM CaCl_2_, for 1 h at room temperature, rinsed in 0.1 M cacodylate buffer (pH 7.2) and then rinsed in distilled water. The cochlear bone is carefully peeled out with a 27-gauge needle, then the organ of Corti is microdissected and the tectorial membrane is pulled out to expose the sensory epithelium. Next, the samples are immersed in a saturated aqueous solution of 1% osmium tetroxide for 1 h in the dark, washed once with water for 10 min, and postfixed with 1% tannic acid aqueous solution for 1 h in the dark (a modified OTOTO protocol). Finally, the samples are rinsed in distilled water.

##### Dehydration in an ascending series of ethanol

Glass scintillation vials (20 ml) are prepared and each is filled with 2 ml of dH_2_O. Each sample is transferred to a vial. The following volumes of absolute ethanol are added to the vial every 10 min: 250 μl, 500 μl, 1000 μl, 2 ml, 4 ml, 8 ml, and 8 ml. Next, the solution is replaced with pure ethanol for 30 min. If samples need to be stored overnight or for extended periods, they have to be kept in 70% ethanol at 4°C to minimize the loss of lipids. However, to achieve the best results we recommend prompt critical point drying.

##### Critical point drying

The top of the mesh basket (EMS, 70190-01) for the critical point dryer is placed into a 60 mm Petri dish filled with absolute ethanol. The samples are carefully transferred into the basket with a glass pipette or a transfer spoon while they are observed under a dissecting microscope. The bottom part of the basket is screwed onto the top part while ensuring the sample remains submerged in ethanol. The sample should never be exposed to the air as ethanol evaporates very quickly and may cause artifacts. Both the top and the bottom parts of the mesh basket (EMS, 70190-01) are made from a stainless steel mesh, which offers good fluid circulation. The mesh baskets with samples are quickly transferred into the critical point dryer chamber filled with ethanol, minimizing the time the sample is exposed to air. Several automated critical point drying systems are available on the market. We recommend using the manufacturer’s protocol for specimen loading and operation. In this work, a Tousimis Autosamdri 815 system was used.

##### Mounting

The appropriate aluminum specimen stub mount must be securely placed under the dissecting microscope to allow for observation and access. Use the specimen stub mount that is compatible with the SEM specimen holder of the microscope. The ultra-smooth carbon adhesive tab (EMS, 77827-12) is placed on the aluminum specimen stub, and the top protective layer is peeled off. The organ of Corti is carefully picked up with the tip of 27G needle and mounted ∼2 mm from the edge of the specimen stub, oriented with hair cells upward and with the cochlea coil facing the edge. The organ of Corti is extremely fragile and delicate after critical point drying, so very small pressure should be applied with the needle to mount it on the specimen stub. To improve the sample conductivity, a small dot of Silver Conductive Adhesive 503 (EMS, 12686-15) can be placed on the carbon disk around the sample if necessary.

##### Sputter coating

The samples are sputter coated with platinum to a thickness of 5 nm using the instrument manufacturer’s protocol. The coated stubs are then transferred to a suitable holder and stored in a desiccator before imaging. The OTOTO (Heywood and Resnick, 1981; Furness and Hackney, 1985) method eliminates the need for metal coating and avoids sample thickening after coating; as a result, the tip links appear much thinner (Furness and Hackney, 1985).

##### Imaging

The samples are observed in a field-emission scanning electron microscope with a SE detector using the SEM manufacturer’s guidelines. To achieve the best orientation of the hair bundles during the imaging and a high SE signal, a 45° tilting sample holder is used (Ted Pella, 15329-7). The aluminum specimen stub mount is oriented with the organ of Corti located at the highest point (closest to the detector). A flat holder for multiple stubs also can be used (Ted Pella, 15310-6) if compatible with SEM. In this study, SEM images were taken with a Hitachi S-4700 field-emission SEM. During the imaging process, the best signal is obtained using the following parameters of the electron beam: accelerating voltage 3–5 kV, current 10 μA, and working distance 6–8 mm.

#### Protocol for Conventional Transmission Electron Microscopy

##### Fixation

P1–P6 explants are prefixed immediately after dissection with 2.5% glutaraldehyde in 0.1 M cacodylate buffer (pH 7.2) supplemented with 2 mM CaCl_2_ for 10 min at room temperature; samples are then transferred to 2.5% glutaraldehyde in 0.1 M cacodylate buffer (pH 7.2) supplemented with 1% tannic acid for 1–2 h at room temperature to visualize links, or to 5% tannic acid for 12 h at 4°C to visualize the stereocilia surface coat. Samples are rinsed three times in sodium cacodylate buffer, 0.1 M, pH 7.4 for 10 min, and then once in distilled water.

##### Post-fixation

For increased contrast, samples are post-fixed with 1% osmium tetroxide/1.5% potassium ferrocyanide in 0.1 M cacodylate buffer for 2 h at room temperature in the dark. Then samples are washed three times in 0.1 cacodylate buffer (pH 7.2), then briefly washed in distilled water.

##### Dehydration

We find most convenient throughout the dehydration steps to keep each cochlea in the 10 ml glass scintillation vials or 1.5 Eppendorf tubes in which cochleas were fixed and stained. The samples are dehydrated at 0°C in an ascending series of ethanol concentrations, as described for SEM, according to the following schedule:

- 25% ethanol two times for 2 min each.
- 50% ethanol two times for 2 min each.
- 70% ethanol two times for 5 min each.
- 80% ethanol two times for 5 min each.
- 95% ethanol three times for 5 min each.
- Absolute ethanol two times for 10 min each.
- Propylene oxide/ethanol (1:1) two times for 10 min each.

Next, samples are equilibrated in propylene oxide three times for 15 min each.

Alternatively, the dehydration can be paused at the 70% ethanol step and the tissue sample can be stored for a day or two in the cold to minimize loss of lipids.

##### Embedding

After the sample is dehydrated, the intermediate solvent such as propylene oxide has to be replaced with a liquid resin that can be polymerized or cured to form a solid block with good sectioning properties.

Samples are infiltrated and embedded in propylene oxide/epoxy resin mixtures as follows:

- 3:1 solution of propylene oxide/epoxy embedding medium for 4–6 h.
- 1:1 solution of propylene oxide/epoxy embedding medium for 4–8 h.
- 1:3 solution of propylene oxide/epoxy embedding medium for 8 h.
- Araldite 502/Embed 812 embedding medium with accelerator for 12 h.

A sufficient amount of embedding resin is added. In each step, the vials are carefully tilted to the sides to ensure that the tissue pieces are fully immersed in embedding resin.

Flat embedding molds (EMS, 70900) are suitable for cochlear samples, as they allow a more accurate orientation of the tissue. Those molds are made from transparent silicon rubber and have elongated recesses 3–5 mm deep. Before transferring the sample into the mold, a small elongated piece of paper is placed with the printed sample identifier (e.g., 06356/p79) opposite the assigned sample location. The molds are filled with Araldite 502/Embed 812 embedding medium with the accelerator. The tissue pieces are transferred into the molds using a plastic inoculating loop. Once the samples are fully immersed in the resin solution, a toothpick is used to align the sample for correct orientation under a dissecting microscope. The samples are polymerized in the oven at 60°C for 48 h.

##### Sectioning

###### Preparation of semi-thin sections (0.7–1.5 μm)

Semi-thin sections can initially be cut, stained with 1% methylene blue/1% sodium borate solution and examined under a light microscope to identify the region of the block containing the organ of Corti and its spatial orientation in the block (Figure 2).

The block is placed into the holder and mounted to the ultramicrotome adapter so the sample is viewed under a dissecting microscope while trimming. A new, single-edged razor blade is used to trim the faces of the block and its edges to form a trapezoid. Unwanted plastic is removed as much as possible. The block is further trimmed with a TrimTool 90 (EMS, Diatome TT-90).

To find the best orientation for sectioning, semi-thin sections are first cut and stained with methylene blue to examine the tissue. The block is sectioned with a diamond knife or glass knife until the tissue is revealed and then a ribbon of 0.7–1.5 μm sections is cut. Several drops of dH_2_O are placed on the glass slide. The sections are transferred onto the water drops and then the slide is placed on a plate preheated to 70–90°C to allow the drops to evaporate and the sections to adhere to the slide. A large drop of methylene blue stain is added onto the sections and the slide is kept on a hot plate for 45–60 s, ensuring that the stain solution does not evaporate. Afterward, the slide is washed with distilled water thoroughly, dried, and examined under a light microscope. The tissue is stained in varying intensities of blue. The sample is mounted with glycerol, covered with a coverslip and observed with a transmitted-light microscope (Figure 2). Depending on semi-thin section results, the block is additionally trimmed or the angle of the trimming is adjusted if the orientation of the tissue is not satisfactory.

###### Preparation of ultrathin sections (60–100 nm)

Before sectioning, the diamond knife edge is checked to be wet, and the level of water is adjusted in the knife boat. An eyelash on a short stick is used to move the water onto the knife edge.

As the 60–100 nm sections are cut and floated on the water, they are seen as silvery in color. Formvar/carbon-coated copper grids with a slot (EMS, FCF2010-CU) are used to pick up sections floating on the surface of the water. The sections are manipulated with the eyelash so that they can be captured with an empty slot grid. The grids are dried by touching a filter paper, then placed in a slotted storage box.

Until recent improvement in diamond knife production, glass knives (ultramicrotomy grade) fitted with appropriately sized plastic knife boats for water reservoirs were the preferred tools for sectioning. It often makes sense for those learning sectioning to start with glass knives before using a diamond knife. The glass knives are sharper than diamond knives, at least for a few minutes, and are far less expensive. Knife makers are commonly found in most EM laboratories.

##### Post staining

Staining should be performed in a closed Petri dish with a dental wax-coated bottom. Ten or twenty grids can be accommodated at once. Before use, uranyl acetate and lead citrate solutions should be centrifuged in Eppendorf tubes at 12,000–14,000 rpm for 3 min.

For uranyl acetate, a Petri dish is prepared with drops of 5% methanolic uranyl acetate. Grids are placed onto drops with the tissue oriented downward, toward the liquid, and incubated for 5 min. Grids are picked individually and rinsed quickly (one dip) in 50% methanol, then twice in dH_2_O (twenty dips each grid). Limit the exposure of uranyl acetate to light during staining. The grids are dried by touching the filter paper. To prevent the formation of precipitates, the grids are thoroughly rinsed and completely dried before beginning the lead citrate staining step.

For lead citrate, a Petri dish is similarly prepared with drops of lead citrate. A few sodium hydroxide pellets are put around these drops. These prevent precipitates when lead solution reacts with CO_2_ from the air. Grids are placed onto drops with the tissue facing downward and incubated for 5 min. Grids are picked individually and rinsed twice in dH_2_O (twenty dips each). The grids are dried by touching a filter paper and placed in a slotted storage box. Once samples are processed according to the protocol described above, the ultrathin sections are examined with a TEM.

##### Imaging

Grids were imaged with a JEOL 1200EX microscope operating at 80 kV. Images were captured with an Advanced Microscopy Techniques camera at 3488 × 2580 pixel resolution.

#### Protocol for Focused-Ion-Beam Scanning Electron Microscopy

The resin blocks for FIB-SEM are made by following steps 1–4 (*fixation, staining, dehydration, embedding*) as described in the section “Protocol for Conventional Transmission Electron Microscopy.”

##### Resin block trimming

As for TEM, the block is placed into the ultramicrotome holder and trimmed using a TrimTool 90 (EMS, Diatome TT-90) to remove unwanted plastic as much as possible. The block is further trimmed with the TrimTool 90 until the embedded tissue is exposed. Semi-thin sections are cut and stained with a methylene blue solution (see section “Preparation of Semi-Thin Sections”), and examined under a light microscope to identify the region of the block containing the organ of Corti with properly oriented rows of inner or outer hair cells.

##### Mounting and sputter coating

Next, the trimmed area of the block is cut off using a new razor blade to a height of about 3 mm. An SEM specimen stub mount compatible with the FIB-SEM microscope is used. The ultra-smooth carbon adhesive tab (EMS, 77827-12) is placed on the aluminum specimen stub, and the top protective layer is peeled off. Next, the trimmed resin sample is carefully picked up with fine forceps and is mounted in the center of the specimen stub, with the working surface facing upward. Silver Conductive Adhesive 503 (EMS, 12686-15) is placed on the carbon adhesive tab around the sample to increase sample conductivity, minimizing charging; then the sample is sputter-coated with 5–10 nm of platinum.

##### Image acquisition, image alignment, segmentation, 3-D reconstruction, 3-D volume analysis

A 3-D serial dataset is obtained on a FEI Helios 660 FIB-SEM microscope using the “Auto Slice and View G3” operating software (FEI). The image acquisition, image processing, and 3-D volume segmentation are performed as previously described (Ivanchenko et al., 2020a).

#### Protocol for Immunogold SEM, Immunogold TEM, Immunogold FIB-SEM

We find it convenient to keep each cochlea throughout the immunolabeling steps in the 1.5 Eppendorf tubes in which they were fixed. It helps to avoid evaporation of the solutions during incubation steps, maintains the concentration of reagents, and significantly reduces the amount of antibody used. During solution exchange, the cochlea remains at the bottom of the tube and the pipet does not touch it. In each step, the tubes are carefully tilted to the side to ensure that the tissue pieces are fully immersed in the solution.

##### Fixation

P1–P3 explants are fixed immediately after dissection with 4% formaldehyde in HBSS with calcium and magnesium, no phenol red (pH 7.2) for 2 h at room temperature. The samples are rinsed three times in HBSS with calcium and magnesium, no phenol red (pH 7.2) for 10 min.

P4–P7 explants are prefixed immediately after dissection with 4% formaldehyde in HBSS with calcium and magnesium, no phenol red (pH 7.2) for 10–15 min, and then transferred to HBSS with calcium and magnesium, no phenol red (pH 7.2). Using fine forceps under a dissecting microscope, the tectorial membrane overlying the hair cells is pulled away to expose the sensory epithelium. The organ of Corti is postfixed with 4% formaldehyde in HBSS with calcium and magnesium, no phenol red (pH 7.2) for 2 h at room temperature. Samples are rinsed three times in HBSS with calcium and magnesium, no phenol red (pH 7.2) for 10 min.

##### Blocking

Samples are blocked in 10% normal goat serum (100 μl per tube) for 2 h at room temperature to reduce background from non-specific binding and Fc receptor binding sites.

##### Incubation with primary antibody

The blocking solution is carefully removed from the tubes and 50 μl of primary antibody is added per tube. The samples are incubated with primary antibodies overnight at 4°C. Extra care is taken in adding each antibody solution to the corresponding labeled tube. Next, they are rinsed three times in HBSS with no calcium or magnesium, no phenol red (pH 7.2) for 10 min.

##### Incubation with secondary antibody

Samples are blocked again in 10% normal goat serum for 30 min at room temperature. The blocking solution is removed and the samples are incubated overnight at 4°C with secondary antibody solution (diluted 1:30 in blocking solution). Following the secondary antibody application, the samples are rinsed three times in no calcium or magnesium HBSS 3x for 10 min each.

##### Post fixation and other steps

For IG-SEM, follow steps 1–4 in the section “Protocol for Conventional Scanning Electron Microscopy.” After samples are mounted they are sputter coated with 5-nm palladium using the coater manufacturer’s guidelines. The stubs are transferred to a suitable holder and stored in the desiccator before imaging. The samples are observed in a field-emission SEM with a backscatter electron detector using the manufacturer’s guidelines. In this study, SEM images were taken with a Hitachi S-4700 field-emission SEM, or FEI Helios 660 FIB-SEM microscope. During the imaging process, the best signal is obtained using the following parameters of the electron beam: voltage 10 kV, current 50 μA, and working distance of 6–8 mm.

For IG-TEM, samples are then processed following steps 1–7 as described in the section “Protocol for Conventional Transmission Electron Microscopy.”

For IG-FIB-SEM, samples are then processed following steps 5–7 in the section “Protocol for Focused-Ion-Beam Scanning Electron Microscopy.”

## Results

**FIGURE 1:**
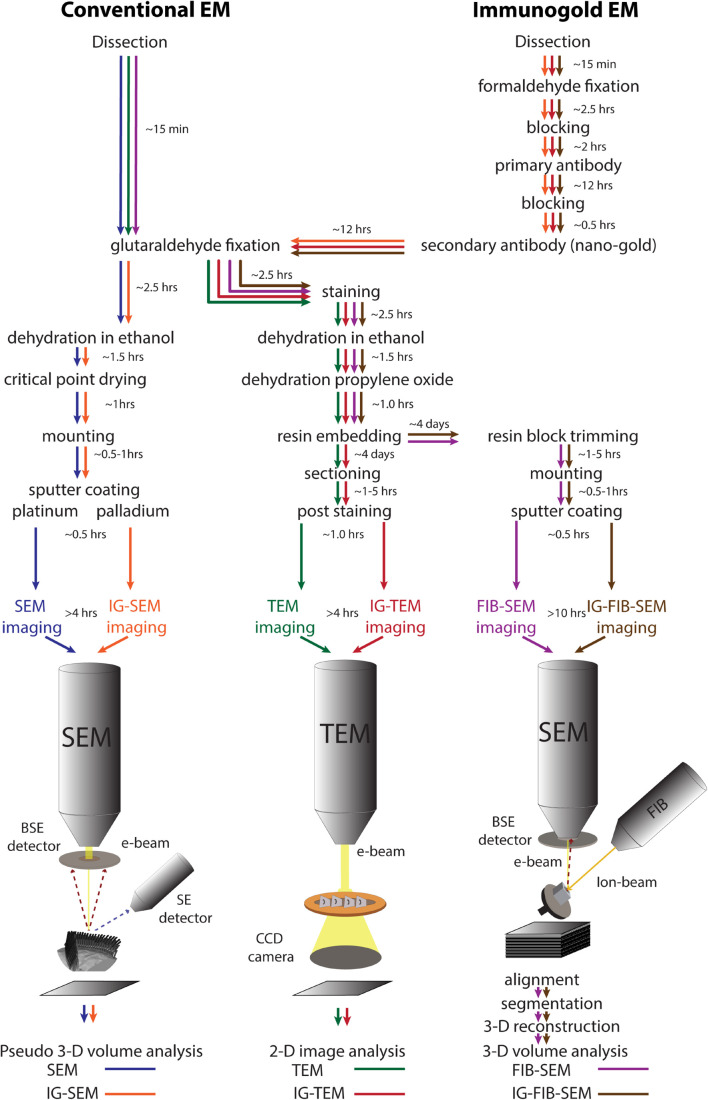
A schematic diagram of the work flow for different electron microscopic approaches: conventional SEM (blue), conventional TEM (green), FIB-SEM (magenta), IG-SEM (orange), IG-TEM (red), IG-FIB-SEM (brown). The time ranges refer to the protocols used in this study. SE, secondary electrons; BSE, backscattered electrons.

Although TEM, SEM, and FIB-SEM use very different imaging systems, the sample preparation for them shares many common steps. These have been developed over many years in our laboratory and others’, and they have been optimized to address the special challenges of imaging stereocilia of the mammalian cochlea and other inner ear organs. Detailed protocols for each are in the section “Materials and Methods,” and are summarized in Figure 1. To illustrate these methods, we describe sequential analysis of hair-cell stereocilia using the different approaches.

**FIGURE 2:**
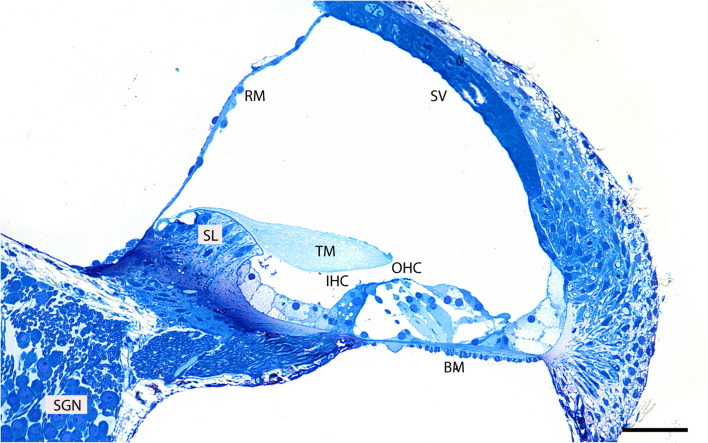
Representative image of a semi-thin (0.5 μm) methylene blue-stained resin section taken from the middle region of an adult mouse cochlea. Basilar membrane (BM), inner hair cell (IHC), outer hair cells (OHC), tectorial membrane (TM), spiral limbus (SL), Reissner’s membrane (RM), spiral ganglion neurons (SGN), stria vascularis (SV). Scale bar 500 μm.

First, to evaluate the gross anatomy of the mouse cochlea and its orientation in the resin block, we analyzed semi-thin plastic sections stained with 1% methylene blue (Figure 2). Clearly visible is the organ of Corti which together with the basilar membrane separates the scala media from the scala tympani. It is composed of mechanosensory hair cells and non-sensory supporting cells.

**FIGURE 3:**
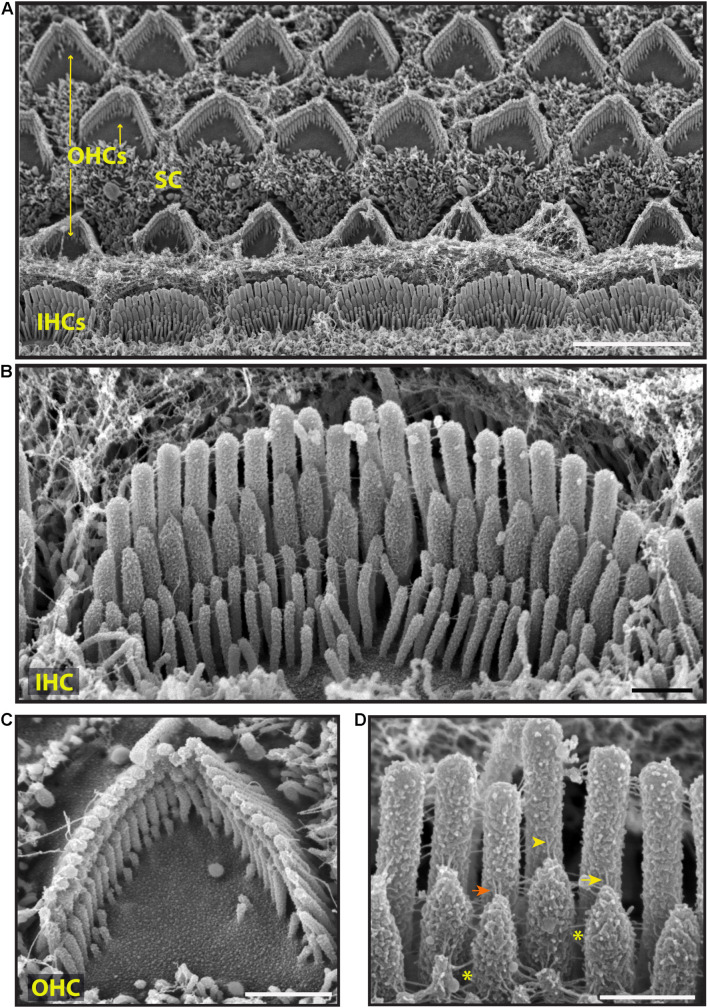
Representative scanning electron micrographs of hair bundles of the neonatal (P5) wild-type mouse cochlea. **(A)** Low magnification micrograph shows an overview of the organ of Corti with three rows of OHCs (yellow arrows), one row of IHCs and supporting cells (SC). **(B–D)** High magnification micrographs of an IHC **(B,D)** and an OHC **(C)** bundles. Tip links connect the tips of adjacent stereocilia along the hair bundle’s axis of sensitivity. These include single tip link filaments (yellow arrow), forked tip links (yellow arrowhead), and “double” tip links from one stereocilium tip (orange arrow). Lateral links (yellow asterisks) spanning the gap between stereocilia are frequently observed. The surface of the stereocilia is rough suggesting the presence of a cell coat material. Samples were sputter-coated with 5 nm platinum and imaged with a field-emission SEM (Hitachi S-4700) using SE detection mode. Scale bars: **(A)** 5 μm; **(B,C)** 1 μm; **(D)** 500 nm.

**FIGURE 4:**
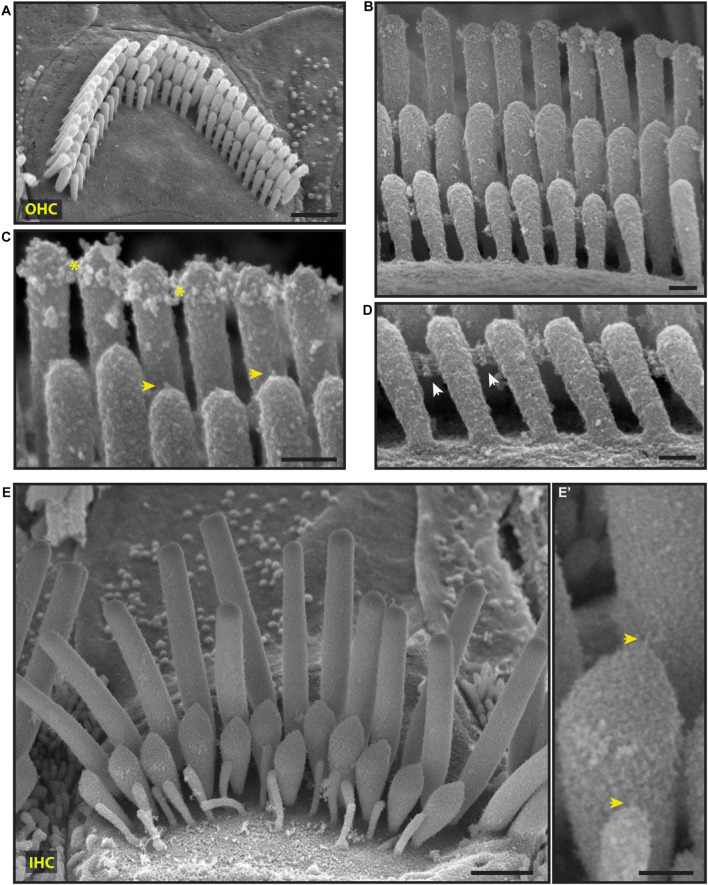
Representative scanning electron micrographs of hair bundles of the adult, wild-type mouse cochlea. **(A–D)** Mature OHC bundles from the middle region of the cochlea. OHC bundles contain tip links (yellow arrows in **C**), tectorial membrane attachment crowns (yellow asterisks in **C**), and horizontal top connectors (white arrows in **D**). The stereocilia surface appears to be relatively smooth suggesting the absence of the cell-coat material. **(E,E’)** Mature IHC bundle from the middle region of the cochlea. Tip links (yellow arrows in **E’**) connect the tips of adjacent stereocilia. The surface of the stereocilia is relatively smooth in appearance which indicates the absence of stereocilia coat. Samples were processed with the modified OTOTO protocol, sputter-coated with 5 nm platinum, and imaged using SE mode. Scale bars: **(A,E)** 1 μm; **(B–D,E’)** 100 nm.

To explore surface specializations of cochlear hair bundles, we used scanning electron microscopy. Conventional low magnification SEM using SE detection in wild-type mice revealed one row of inner hair cells (IHCs) and three rows of outer hair cells (OHCs), separated by supporting cells (Figure 3A). Both types of hair cells have actin-filled stereocilia at their apical surfaces in rows of increasing height (Figures 3B,C, 4). At this age (P5), stereocilia are cross-linked by a variety of links. Conventional SEM of neonatal cochlea showed that tip links connected the tips of adjacent stereocilia along the hair bundle’s axis of sensitivity (Figures 3D, 4B–E’). These tip links include single filaments, forked filaments, and “double” tip links which have been described previously in developing cochleas (Alagramam et al., 2011). Lateral links connecting adjacent stereocilia are frequently seen (Figures 3B,D). The surface of the stereocilia is rough indicating a cell coat material or glycocalyx.

Scanning electron microscopy of adult cochlea showed well-organized hair bundles in IHCs and OHCs (Figure 4). In OHC bundles the stereocilia surface appeared to be relatively smooth suggesting the absence of the cell-coat material at that age (Figure 4). Horizontal top connectors connected the stereocilia within a single row (Figures 4B–D) and between adjacent rows. Tectorial membrane attachment crowns were observed on the tallest row of OHC stereocilia (Figures 4B,C). The adjacent stereocilia of both IHCs and OHCs were interconnected by tip links formed by PCDH15 and CDH23 proteins (Kazmierczak et al., 2007; Figures 4B,C,E’).

**FIGURE 5:**
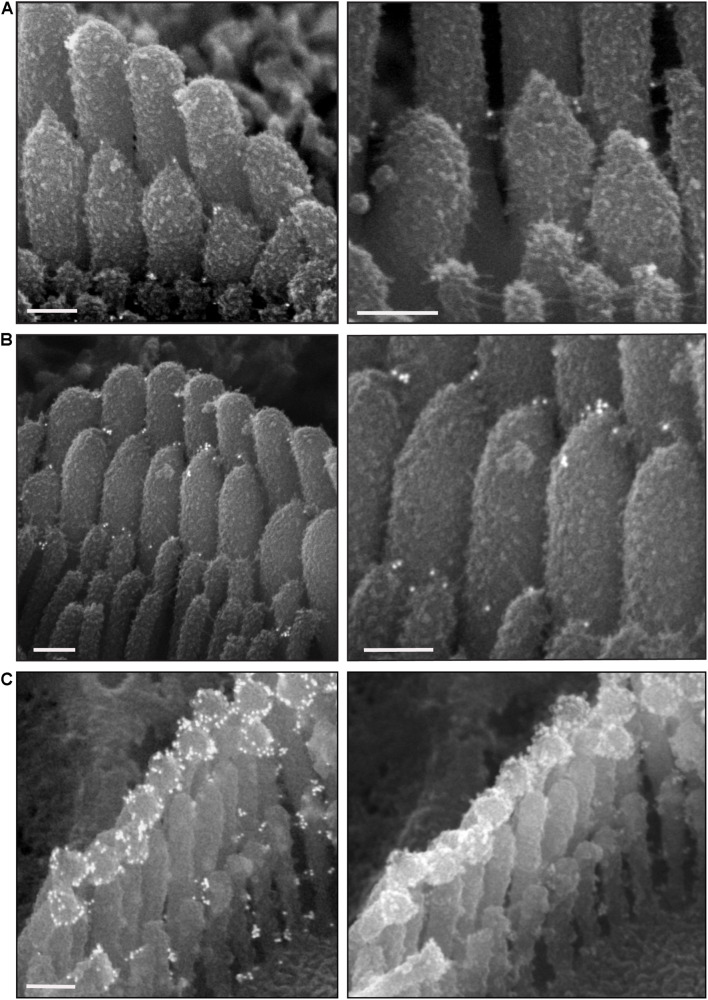
Representative immunogold scanning electron micrographs of OHC hair bundles of the neonatal (P6) wild-type mouse cochlea. Samples in **(A,B)** were immunostained with anti-PCDH15 primary antibody and gold-conjugated secondary antibody, sputter-coated with 5 nm palladium, and imaged with backscatter electron detectors on different microscopes. **(A,B)** Were prepared in parallel. Gold beads (12 nm) localize mostly at the tips of shorter-row stereocilia. **(A)** Sample imaged with a field-emission SEM (Hitachi S-4700) using BSE detection mode (semiconductor BSE detector). Right panel is higher magnification. **(B)** Sample imaged with a field-emission FEI Helios 660 FIB-SEM using BSE detection mode (high contrast solid-state BSE detector, CBS). Right panel is higher magnification. **(C)** Sample was immunostained with anti-STRC primary and gold-conjugated secondary antibodies, using a protocol similar to that in methods, and imaged (left) with a Helios 660 FIB-SEM using BSE or (right) with a Hitachi S-4700 using SE detectors. Scale bars: **(A–C)** 200 nm.

To identify proteins associated with these specializations, we used immunogold SEM. We first processed neonatal (P6) wild-type mouse cochlea immunostained with anti-PCDH15 primary antibody and colloidal gold-conjugated secondary antibody. We acquired images with SEM using the backscatter electron detector to distinguish the gold beads from the sputter-coated palladium on the surface. Gold beads associated with anti-PCDH15 were observed at the tips of stereocilia and often were associated with the tip links (Figures 5A,B). We also stained cochleas with anti-stereocilin (STRC) antibodies (Figure 5C). STRC is thought to participate in the TM attachment crown and perhaps horizontal top connectors (Verpy et al., 2001, 2011). In a secondary electron image (Figure 5C, right), knobby structures observed at the tips of the tallest stereocilia represent the tectorial membrane attachment crowns (see also Figures 4B,C). In a backscatter image of the same bundle (Figure 5C, left), gold beads bound to anti-STRC antibodies decorated the knobs, indicating that the attachment crowns are composed in part of STRC.

**FIGURE 6:**
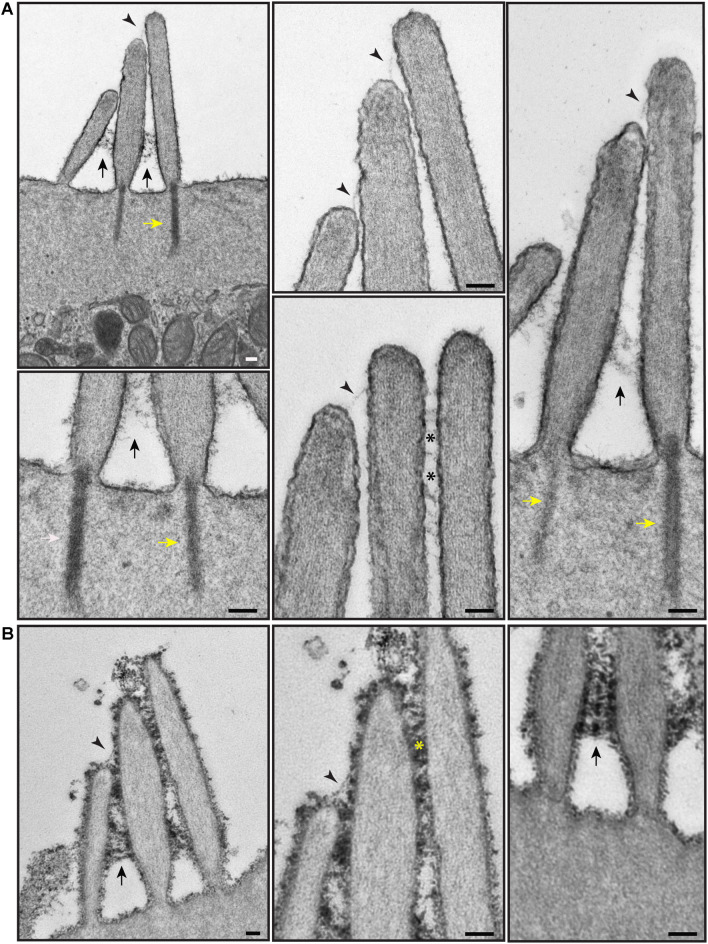
Representative transmission electron micrographs of OHC hair bundles of the neonatal (P4) wild-type mouse cochlea. **(A)** Samples stained with 1% tannic acid (1 h). **(B)** Samples stained with 5% tannic acid (overnight). In **(A,B)** adjacent stereocilia (short, middle, tall) are seen to be connected by tip links (black arrowheads), lateral links (asterisks), and ankle links (black arrows). Yellow arrows point to the rootlets. The cell coat material is better visible on the samples stained with 5% tannic acid. It appears at the surface of stereocilia and the apical non-stereociliary surface. **(A,B)** Samples were imaged with a JEOL 1200EX microscope operating at 80 kV. Scale bars: **(A,B)** 100 nm.

To better visualize actin within hair cells, the surface coat on the apical surface, and extracellular links, we used TEM. We used two TEM sample staining protocols on P4 neonatal wild-type mouse cochleas—either a brief 1% tannic acid treatment to highlight the links and actin filaments (Figure 6A), or an overnight 5% tannic acid treatment to highlight the surface coat (Figure 6B). Actin filaments were observed in the stereocilia, the rootlets, and the cuticular plate (Figure 6A; Tilney et al., 1980; Pacentine et al., 2020). Stereocilia were filled with parallel electron-dense actin filaments. Actin-containing rootlets anchored stereocilia to the cell body. The cuticular plate contained a network or mesh of actin, made up of filaments with no specific orientation or directionality. Cell surface specializations such as tip links, ankle links, and transient lateral links were observed on the hair bundles of OHCs (Figures 6A,B). On samples stained with 5% tannic acid, an electron-dense cell coat was seen over the entire stereociliary surface and cell membrane over the cuticular plate (Figure 6B).

**FIGURE 7:**
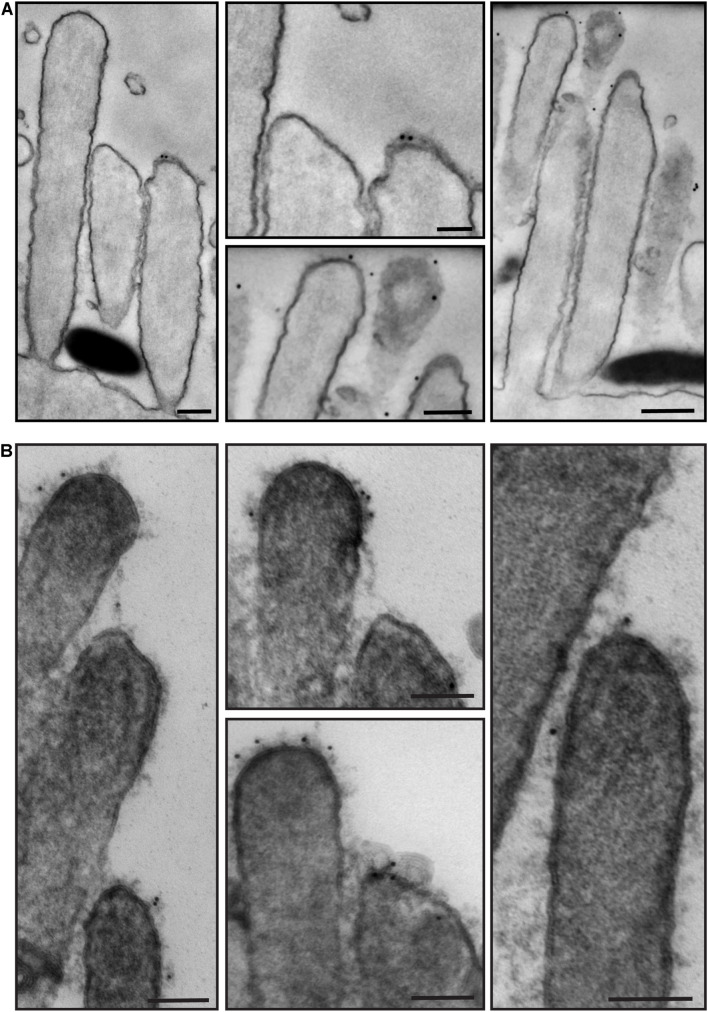
Representative immunogold transmission electron micrographs of OHC hair bundles in neonatal (P4) wild-type mouse cochlea. Samples immunostained with anti-PKHD1L1 primary antibody and gold-conjugated secondary antibody. Gold beads (10 nm) localize near the tips of stereocilia. **(A,B)** Were prepared in parallel, however in **(B)** post-staining with uranyl acetate and lead citrate significantly increased contrast of stereocilia cores and surface coat. **(A,B)** Samples were imaged with a JEOL 1200EX microscope operating at 80 kV. Scale bars: **(A)** 200 nm; **(B)** 100 nm.

We then processed for TEM samples that had been immunostained with anti-PKHD1L1 primary antibody and colloidal gold-conjugated secondary antibody. PKHD1L1 participates in forming the stereocilia surface coat, causing hearing loss when absent from the hair cells (Wu et al., 2019). Anti-PKHD1L1 10-nm gold beads were localized toward the tips of OHC stereocilia (Figures 7A,B) and were often associated with the surface coat, which was seen as a dense, uniform fuzz on the membrane (Figure 7B). No gold beads were associated with the tip links or the transient lateral links connecting neighboring stereocilia, suggesting that PKHD1L1 does not participate in forming links between stereocilia but may attach stereocilia to other structures.

**FIGURE 8:**
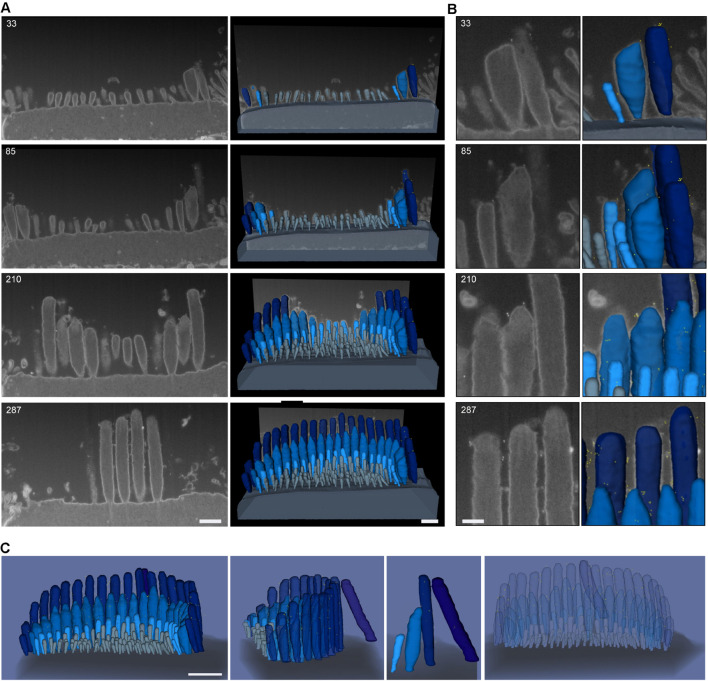
Representative FIB-SEM of IHC hair bundles of neonatal (P1) mouse cochlea. Samples were immunostained with anti-PCDH15 primary antibody and gold-conjugated secondary antibody (12 nm gold). **(A)** *Left*, representative FIB-SEM micrographs from four single planes; *right*, a 3-D rendering of the FIB-SEM image stack. **(B)** An inset from **(A)** showing a closer view of the stereocilia and gold beads at their tips. Sample was imaged with a field-emission FEI Helios 660 FIB-SEM using BSE detection mode. **(C)** 3-D representation of an IHC stereocilia bundle labeled with anti-PCDH15 immunogold (12 nm) (426 serial FIB-SEM cross sections, at 10 nm milling step; yellow, gold beads; blue, stereocilia; light gray, microvilli; dark gray, cell body). At this age, PCDH15 is diffusely located near stereocilia tips. Scale bars: **(A)** 500 nm; **(B)** 200 nm; **(C)** 1 μm.

To evaluate the distribution of the tip-link protein PCDH15 at the surface of stereocilia, we used FIB-SEM to collect serial EM data sets of neonatal (P1) IHC stereocilia immunostained with anti-PCDH15 primary antibody and colloidal gold-conjugated secondary antibody (12 nm gold). Serial data sets were collected, objects of interest were identified and outlined based on image contrast contours (segmented), and objects were 3-D reconstructed using Dragonfly and Amira software packages (Figure 8). These revealed that in neonatal cochlea, PCDH15 was localized at the tips and along the surfaces of the stereocilia, suggesting that it was a component of tip inks and transient lateral links (Figure 8 and Supplementary Movie 1).

## Discussion

In this study, we aimed to describe and compare different EM imaging techniques commonly used to study hair cells of the inner ear. Although the EM methods have been used for years, various efforts to improve the outcomes have been undertaken. Step-by-step, we described conventional and immunogold TEM, SEM, FIB-SEM providing practical advice for both beginners and experts, and incorporated many years of personal experience in using these approaches in the inner ear research. In our experiments, we used mouse cochlear tissue, however, these protocols with minor modifications also can be applied to the inner ears of other species.

### Specimen Preparation for Electron Microscopy

The introduction of artifacts is one of the most common problems in EM, which makes it difficult to judge whether a cell’s ultrastructure is properly represented in the final images. Specific indications of possible artifacts include distortion and disorganization of hair cell bundles, loss of cell-surface specializations, loss of continuity and irregularity of the cell membranes, disparity in the preservation of adjacent cells of the same type, swollen and empty spaces in the cytoplasm and the perinuclear space, and disorganization of the organelles and filamentous structures. Several additional criteria may be used to evaluate the presence of artifacts. First, if the final appearance of the sample conflicts with previously published ultrastructure of the tissue, it is more likely an artifact than an important new discovery. Second, images should be consistent with those biochemical and physiological data that have structural correlates and are well described. Thus characterizing how a genetic mutation affects ultrastructure is especially difficult as mutational effects could be confused with artifacts from sample preparation. In practice, the best test is a comparison of the mutant sample with a control sample of normal morphology prepared in parallel. Following recommendations available in the literature (Bozzolla and Russell, 1998) and protocols described by other groups (Reynolds, 1963; Heywood and Resnick, 1981; Pickles et al., 1984; Furness and Hackney, 1985; Glauert and Lewis, 1998; Verpy et al., 2001, 2011; Goodyear et al., 2005; Michel et al., 2005; Ahmed et al., 2006; Harris et al., 2006; Kazmierczak et al., 2007; Grillet et al., 2009; Fischer et al., 2012; Indzhykulian et al., 2013; Kizilyaprak et al., 2014; Fang et al., 2015; Miranda et al., 2015; Jones, 2016; Parker et al., 2016; Velez-Ortega et al., 2017; Velez-Ortega and Frolenkov, 2019), we used fixation, staining, immunolabeling, dehydration, embedding reagents, and conditions that minimize these factors.

#### Cochlear Dissection

A critical first step of sample preparation is cochlear dissection. Hair cells with their rows of fine stereocilia are very delicate structures and the integrity of hair cell bundles can be easily disrupted during dissection. Researchers must perfect their cochlear dissection technique before carrying out meaningful electron microscopy experiments. The dissection and most processing steps should be performed under the visual control of a dissecting microscope. To assess preservation of structure during dissection, light microcopy of samples in which actin is stained with phalloidin can be helpful in preliminary experiments (Landegger et al., 2017). The removal of the tectorial membrane is also problematic for the integrity of hair cells; it requires steady hands, proper tools, and experience (Landegger et al., 2017). In TEM a tectorial membrane can usually be kept in place, in SEM in adults, the dehydration nearly always lifts the tectorial membrane from the stereocilia and it curls back to reveal the tops of the hair cell as well as stereocilia imprints on the surface of the tectorial membrane. It goes further up and back after critical point drying. However, in SEM in neonates or any EM that involves antibody labeling, it is important to fully expose the sensory epithelium. This problem is eliminated in post-embedding labeling. We found that slight prefixation of the sample with 4% formaldehyde for 10–15 min makes the tectorial membrane removal easier and minimizes damage to the stereocilia in this step.

Transfer of the samples is also particularly important; ideally, the sample should always be immersed in liquid and the sensory epithelium should never touch the surface of the pipet or other instruments. Finally, the dissection time should be as short as possible. Even though the dissection medium (often Leibovitz’s L-15 medium) is enriched with amino acids, vitamins, inorganic salts, and other components to maintain cell health and survival in environments without CO_2_ equilibration, there is a risk of autolysis. Autolysis causes not only bad preservation of ultrastructure but may also destroy antigenic sites and cause false-negative immunogold results (Jones, 2016). The best results are obtained if the fixative reacts with the sample within seconds after the removal from the cochlea.

#### Fixation

Fixation is also one of the most important steps in sample preparation for EM. The goal of fixation is to stabilize cellular organization enough that ultrastructural relationships are preserved despite the subsequent rather drastic treatments of dehydration, embedding, and exposure to the electron beam (Riemersma, 1968). Aldehydes are the most widely used fixatives in EM. For optimal preservation of ultrastructure, a relatively strong glutaraldehyde fixative at a concentration of 2.5% is the primary choice. It contains two functional aldehyde groups which react with cellular components, especially with the amino groups in proteins, forming cross-links and resulting in good preservation of ultrastructure. In EM, tannic acid is a useful addition to aldehyde fixation. It does serve as a fixative but has very slow penetration and needs to be used in combination with another fixative such as glutaraldehyde or osmium tetroxide (Glauert and Lewis, 1998). Besides its fixative properties, it enhances the electron density of heavy metals (osmium tetroxide, uranyl acetate, and lead citrate) resulting in increased contrast of extracellular structures, such as the surface coat and stereocilia links. It is less effective for staining intracellular structures but still helps to reveal actin filaments of stereocilia.

Some antigens are extremely sensitive to cross-linking by glutaraldehyde, and this fixative can compromise antibody labeling. For immunogold experiments, formaldehyde is used as a primary fixative as it is a more “gentle” fixative and keeps epitopes accessible for antibodies. The cost is the inevitable loss of some ultrastructural details. For optimal preservation of both immunoreactivity and cell ultrastructure, the formaldehyde fixation step is followed by post-fixation with glutaraldehyde after immunolabeling is completed. Specific attention should be paid to fixation time and temperature. Over-fixation and under-fixation can lead to false-negative or false-positive immunolabeling. The temperature during fixation should be close to the same as when the cochlea was dissected, since changes in temperature can affect the surface morphology of the sensory epithelium. In our experiments, these steps are usually done at room temperature.

For small samples such as neonatal cochlear explants, immersion in the fixative is a preferred method. For adult cochlea, in which the organ of Corti microdissection is more challenging and time-consuming, intracardial perfusion is often a preferred method of primary fixation. However, we did not observe significant differences in ultrastructure when a whole cochlea was quickly dissected out from the temporal bone and moved to the fixative in less than a minute. For this procedure the cochlea is gently and slowly perfused with 4% formaldehyde/1% glutaraldehyde via the round and oval windows until the solution was washed out of the small hole at the apex; it is next postfixed with 2.5% glutaraldehyde. Karnovsky’s fixative (2% formaldehyde/2.5% glutaraldehyde) can be used as an alternative solution. Thereafter, the microdissection is performed in the already fixed sample. The advantage of using formaldehyde/glutaraldehyde or Karnovsky’s fixative is based on the ability of formaldehyde to penetrate more rapidly to stabilize the cell structure, and glutaraldehyde simultaneously but more slowly permanently cross-link proteins.

#### Buffers

Besides the selection of the appropriate fixative, it is important to use the right buffer. Traditionally the buffers used for EM are designed to have a similar osmolality to native tissue (300 mOsmol). Phosphate buffer is widely used because it mimics the composition of extracellular fluid and is not toxic to cells. However, it has a number of practical disadvantages. The most important is that it is incompatible with calcium, in that calcium phosphate forms a precipitate which can be very visible on the surface of stereocilia. Also, calcium ions are needed to preserve stereocilia links. Finally, stock phosphate buffer solution can be easily contaminated with microorganisms. None of these apply to cacodylate buffer, which is the favored buffering solution used with fixatives in specimen preparation for electron microscopy. Cacodylate buffer prevents microprecipitation, and ultrastructural preservation is excellent. For the samples processed for immunogold labeling, HBSS is the preferable buffer in the fixation and immunolabeling steps. As discussed above, formaldehyde causes gentle fixation and HBSS helps to maintain physiological pH and osmotic balance in under-fixed cells. It also provides essential inorganic ions, such as calcium, to preserve ultrastructural details during immunostaining steps.

#### Staining

In order to postfix and enhance the contrast of the cell ultrastructures, samples are usually stained with osmium tetroxide, often in combination with potassium ferrocyanide. The osmium tetroxide slowly reacts with proteins, including histone proteins, helping to preserve the associated DNA (Bozzolla and Russell, 1998). It also postfixes and stains phospholipids of the cell membrane and organelles. The process of interaction between osmium and potassium ferrocyanide is poorly understood but observations show that glycogen staining is enhanced by post-fixation with osmium tetroxide in the presence of ferrocyanide: there is usually more staining of cell-surface specializations and cell membranes (Wu et al., 2019). However, osmium stops the activity of enzymes in the cell which leads to cell hardening. Samples become extremely fragile and can be easily damaged by rough pipetting. The use of osmium tetroxide is additionally problematic for immunolabeling because it results in complete loss of antigenicity. Thus, it has to be applied when samples are already immunogold labeled. In some post-embedding immunogold techniques, osmium was used and good labeling still had been achieved (Flechsler et al., 2020).

#### Dehydration

Since most embedding media are not soluble in water, it is necessary to dehydrate fixed samples by passing them through a sequence of dehydrating solutions that are fully soluble with the embedding medium. The most broadly used dehydrating agents are ethanol, acetone, and propylene oxide (Bozzolla and Russell, 1998). The major problem of dehydration is the shrinkage of the cells as a result of the extraction of lipids and water from the sample (Bozzolla and Russell, 1998). A gradual increase in ethanol concentration prevents tissue shrinkage during the dehydration step. The following factors, which minimize the loss of lipids during the dehydration step, should be considered: (1) maintaining calcium ions in the fixative and washing solutions; (2) postfixation with osmium tetroxide and potassium ferrocyanide; (3) shorter duration for dehydration steps; (4) processing at 0°C; and (5) use of gentle lipid solvents such as ethanol, acetone.

#### Embedding, Trimming, Sectioning, and Post-staining

In TEM or FIB-SEM the dehydrated sample should be infiltrated with an epoxy resin which is then polymerized to a solid block with good sectioning properties. Epoxy resins (e.g., Araldite 502/Embed 812, Epon 812, Spurr’s resin, Durcupan) have considerable advantages as embedding media for EM, in comparison with the acrylic resins. Their volume changes very little during polymerization, they harden uniformly, they have good sectioning properties, and they are very stable under an electron beam. Acrylic resins (Lowicryls, LR-White) are transparent and have a low viscosity as the temperature is lowered, so specimens can be infiltrated and the resin can be polymerized at low temperature. Additionally, LR-White is a hydrophilic acrylic resin and can be used for post-embedding immunolabeling techniques.

For TEM, individual ultrathin sections, 60*–*100 nm in thickness, are sectioned using an ultramicrotome with a diamond knife and are picked manually onto a metal support grid. We use copper formvar/carbon-coated grids with a slot, and a transparent support film suspended across the slot. These grids allow us to mount the sections as flat as possible on support materials. Grids for TEM can be post-stained with heavy metals that significantly enhance electron-scattering, resulting in increased contrast in the electron microscope (Figures 6, 7). But post-staining of ultrathin sections is not absolutely necessary and samples can be imaged without it (Figure 7). We recommend keeping some grids unstained as a backup.

In FIB-SEM the resin block with the sample is trimmed using an ultramicrotome until the embedded tissue is exposed, then it is mounted on the specimen stub oriented with the working surface upward. Silver conductive adhesive is placed on the carbon adhesive tab around the sample to increase conductivity and minimize charging, and the sample is sputter-coated with 5–10 nm of platinum.

#### Critical Point Drying

During SEM sample preparation the absolute alcohol of the dehydrated sample needs to be removed, but the surface tension of the alcohol can distort the tissue if air-dried. To allow drying, the alcohol is replaced with liquid CO_2_ in a critical point dryer. At the right temperature and pressure (the “critical point,” 31.1°C and 73.8 bar), the gas and the liquid phases become identical and the visible boundary between them vanishes, reducing surface tension to zero, resulting in instant evaporation of CO_2_. The structure of the hair bundles is expected to remain largely intact after the gas has been slowly released from the chamber. The dried sample is then mounted on the specimen stub covered with a carbon adhesive tab, and sputter coated. To improve the sample conductivity, silver colloidal paint can be used but is more difficult to apply, especially with small samples.

#### Sputter Coating

The coating of SEM samples with a conductive metal is a required step to optimize the microscope performance during imaging. It allows non-conductive SEM samples to become conductive so that the charge of incident electrons is carried away. Otherwise the electric field created by a charged sample would distort the incident electron beam. Coating thus enables higher sample stability under the electron beam and better resolution. During our protocol development for conventional SEM, we have tested chromium, cobalt, aluminum, copper, carbon, platinum, and palladium coating materials, and found that a 5 nm coating with platinum gives an acceptable resolution of fine stereocilia structures such as tip links and other cell surface specializations. For immunolabeling with colloidal gold samples, we instead use a 5 nm coating with palladium, as previously reported (Indzhykulian et al., 2013). Platinum has nearly the same atomic number (78) as gold (79), and so antibody-coupled gold beads cannot easily be distinguished from a platinum coating using the energy of the BSE. Palladium has a much lower atomic number (46); it allows sufficient contrast with gold while also offering good resolution. Other coating materials have also been used, including carbon (Goodyear et al., 2010). It is also possible to observe immunogold particles on samples prepared with the OTOTO technique (Heywood and Resnick, 1981; Osborne and Comis, 1991; Han et al., 2020), which is sometimes necessary in adult cochlear samples. However, while OTOTO reduces charging, osmium (atomic number 76) partially masks the gold, making the detection more challenging.

The sensory epithelium is not flat, but is a convoluted surface with stereocilia bundles angled to the basilar membrane. The uniform deposition of coating material on the surface of the stereocilia is very important for good conductivity but not possible if the deposition from the target material is perpendicular to the specimen stub surface. The samples should be tilted and rotated to achieve a continuous coating. Most sputter coaters can be equipped with such “planetary” rotating platforms. In our hands, the best result is achieved when the deposition of the palladium or platinum occurs when the stage with the sample is rotating and tilted 35° toward the target for two-thirds of the total deposition time, and 10° away from the target for one-third of the total time.

#### Immunogold Labeling

Immunogold EM was historically more often used with TEM. However, with new methods, it has become possible to apply these techniques in SEM. Most developed protocols include specific labeling with a primary antibody, either directly conjugated to gold beads (direct labeling) or subsequently labeled with a secondary antibody conjugated to gold beads (indirect labeling). Primary antibodies can be monoclonal or polyclonal. Polyclonal antibodies bind to multiple epitopes and are more sensitive, while the monoclonal antibodies recognize a single epitope, thereby being more specific (Magaki et al., 2019). A primary antibody should be raised in a different species from the species in which it is used, to avoid cross-reactivity (Boykins et al., 2016). It is also necessary to have a well-characterized antibody that can efficiently recognize the epitope after formaldehyde fixation.

In this work, we used indirect labeling, where the primary antibody is unconjugated, and labeling of the primary antibody is achieved with a gold-conjugated secondary antibody. The advantage of the indirect procedure is that a variety of commercial secondary antibodies are available, reducing the cost. The main disadvantage is that the gold beads are located anywhere within a radius of ∼10–15 nm around the epitope site, due to the length of antibodies.

The concentration of antibody that will provide the strongest staining of the target antigen and lowest background staining must be determined by serial dilutions of a concentrated antibody. It is advisable to start with the dilution recommended by the manufacturer, and also consider one dilution 2–3-fold above it and one dilution below (Magaki et al., 2019).

Double labeling for two proteins can be achieved with EM if the two primary antibodies are raised in different species, so the respective secondary antibodies can differentially recognize them, and if the secondary antibodies are conjugated to colloidal gold of different sizes (e.g., 6-nm, 10-nm, 12-nm, and 18-nm gold). Extensive controls are absolutely essential for the analysis of the results. Some level of non-specific binding (background) is always possible but it is important to minimize it.

In this study, we developed a pre-embedding procedure for proteins that are expressed extracellularly on the surface of the stereocilia. The protocol uses HBSS during the immunolabeling steps, and an additional post-labeling fixation step with glutaraldehyde to minimize ultrastructural loss. For TEM and FIB-SEM samples, in addition to post-fixation with glutaraldehyde, we applied tannic acid and osmium tetroxide to stabilize the antibody complexes and to prevent ultrastructural degradation that appears during the dehydration and heating steps associated with embedding in epoxy resins.

### Electron Microscopy Techniques

There is no single recommendation on which approach or tool is the best choice to answer a specific biological question. More and more techniques are becoming available, and each has its advantages and limitations regarding the resolution, sample area, volume to be analyzed, time for sample preparation and imaging, difficulties in microscope operation which are usually the main factors to be considered when a decision to use one or other approach has to be made (Table 1).

**TABLE 1:** Key parameters of different EM methods.

	TEM	IG-TEM	SEM	IG-SEM	FIB-SEM	IG-FIB-SEM
**Equipment**	Fume hood, dissecting microscope, ultramicrotome, light microscope, TEM (80–100 kV)		Fume hood, dissecting microscope, critical point dryer, sputter coater, SEM with BSE detector		Fume hood, dissecting microscope, ultramicrotome, light microscope, sputter coater, FIB-SEM	

**Time for sample preparation**	5 days	7 days	7–24 h	2–3 days	5 days	7 days

**Measurements**	2-Dimensional		Pseudo 3-Dimensional		3-Dimensional	

**Achievable resolution**	Transmitted electrons *x*, *y*: ∼0.2–0.5 nm, z: 60–100 nm (limited by section thickness)		Secondary electrons *x*, *y*: ≥0.5 nm	Backscattered electrons *x*, *y*: ≥2 nm	Backscattered electrons *x*, *y*: ∼ 2 nm, z: ∼5–20 nm	

**Dimensions**	*x*, *y*: <50 μm (more is possible but not reasonable)		*x*, *y*: <50 μm		*x*, *y*, *z*: ∼ 20 μm (more is possible but not reasonable)	

**Non-destructive/Destructive**	Non-destructive approach		Non-destructive approach		Destructive approach	

**Preinspection of the area of interest**	Possible		Possible		Not possible in detail until the milling process is started	

**Imaging time**	- Several hours and more - Time depends on user experience and quality of the samples - A number of manual adjustments are needed - Structures of interest in each section have to be selected by the user				- Tens of hours and more, depending on the volume - Time depends highly on experience - A number of manual adjustments are needed - No need to select individual profiles of the same cell - The cell is sequentially sectioned and imaged in an automated fashion	

**Application**	Sections of hair cell including stereocilia, kinocilia, general organelles, cell nucleolus, membrane, cell surface specializations (links, coat)	Same as TEM plus individual proteins immunolabeled with gold beads	Hair bundle surface topography, stereocilia links	Same as SEM plus cell-surface proteins immunolabeled with gold beads	-Visualization and 3-D reconstruction of entire hair cell or area of hair cell including stereocilia, kinocilia, organelles, cell nucleolus, membrane -Visualization and reconstruction of links is possible but challenging	
						Individual proteins immunolabeled with gold beads

#### Conventional and Immunogold Transmission Electron Microscopy

Transmission electron microscopy historically was a preferable method for ultrastructural examination of hair cells based on its sub-nanometer resolution. Deposition of heavy metals such as uranyl acetate and lead citrate onto cellular structures increases the contrast but reduces the resolution. The grids are then inserted into the microscope and a small area of interest (10–20 μm) is selected for imaging. Accelerated electrons pass through the sample and are focused onto a CCD camera detector, with 0.2–0.5 nm pixel size. Scattering of the electrons by heavy metals in the sample generates dark regions in the resulting image. TEM imaging is parallel in nature, with each pixel on the image corresponding to a relevant location within the focal plane of the imaging area and all pixels are collecting electrons simultaneously. TEM is a non-destructive technique and the specimens can be imaged multiple times for larger field sizes or at higher resolution.

Beam alignment, stigmation, and focusing are basic settings that affect the quality of a TEM image. These have to be adjusted by the operator, at magnifications higher than used for imaging. The optimal parameters depend widely on the application. Generally, higher voltages, smaller apertures, and smaller spot sizes make the resolution higher. However, compromise often is necessary to optimize the settings. Astigmatism and spherical and chromatic aberrations are all factors that can affect imaging quality. Of course, a perfectly aligned microscope is essential for generating high-quality data.

Transmission electron microscopy also has some disadvantages. The embedding preparation of biological tissue means the resolution 0.2–0.5 nm is not easily achievable because of limitations such as the molecular graininess of the polymerized resin, the “fuzziness” of extended biological molecules, and the additional coatings added by staining. However, certain applications allow a high resolution to be achieved (e.g., negative staining). Manual sectioning for TEM often generates artifacts in the sections such as holes, folds, shrinkage, and stretching. There is also limited control of the sectioning plane. Staining steps may result in precipitation of heavy metals, which can cause holes in the formvar film or loss of sections. The success of TEM very much depends on the skill of the operator, and on accuracy in collecting and imaging each ultrathin section. Only a relatively small area can be imaged in detail, and low penetration of the electron beam and aberrations limit samples to a thickness of 60–100 nm. Also, TEM provides only two-dimensional information unless multiple serial sections are collected, imaged and reconstructed into a 3-D volume. On the other hand, TEM imaging does not require specialized equipment beyond that common to a basic EM laboratory and is therefore relatively inexpensive.

#### Conventional and Immunogold SEM

Interpreting the two-dimensional images of TEM micrographs is challenging, especially in understanding the plane of the section through the tissue. Thus SEM is better at representing a sample with a complex surface. SEM images are formed by the detection of SE or BSE that are emitted when the electron beam hits the surface of the sample. SE are ejected from atoms of the sample by inelastic collision by beam electrons, and collected by a detector placed to the side. Contrast is produced by a difference in emission when the beam hits the surface at varying angles, creating the sense of shadow in the image. These images provide detailed topographic information with a resolution close to 0.5–1 nm. Secondary electron imaging is an ideal mode with which to investigate a complex structure like a cochlear sensory epithelium, and to assess hair cell number; bundle morphology; planar cell polarity; shape, length, width of stereocilia; and stereocilia links. BSE are generated when incident beam electrons are elastically scattered by atoms in the sample back toward the source, and are collected by a donut-shaped detector concentric with the incident beam. Backscatter electron generation depends on the atomic number of atoms in the sample and so can distinguish different metals. Backscatter imaging is typically used for immunogold SEM, to create strong contrast of the gold beads labeling the tissue.

Biological samples are composed of low atomic number elements and when the electron beam interacts with the sample, electrons can penetrate deeply, causing loss of signal and decrease in sample resolution. Appropriate coating or metal impregnation, and adequate imaging settings should be considered to optimize the imaging conditions. Generally, an electron beam of lower voltage is more suitable for biological samples because it does not penetrate into the sample as deeply as with higher accelerating voltage; however, it produces a weaker signal and lower instrument resolution. For 5 nm platinum-coated samples, 3–5 kV provides adequate secondary electron signal and good resolution of surface features such as stereocilia links (Figure 3). A higher accelerating voltage (10 kV or more) may result in a poor image due to deep penetration of the electron beam and high charging of the samples. Charging is a phenomenon that gives rise to anomalous contrast due to the fact that the amount of the electrons emitted from the specimen is larger than the incident electrons in some locations of the specimen surface and is commonly observed at low conductive specimens. Notably, tip links are not well resolved by high-voltage electron beams because the high-energy electrons pass through them instead of scattering off them; a low-voltage (∼5 kV) beam is needed. When a low accelerating voltage is used, instrument resolution can be retained by use of a field-emission electron source.

Samples processed with the osmium-based OTOTO method (Figure 4) can be imaged at 3–10 kV with good resolution, low charging, and good stability of the stereocilia under the beam. This protocol works better in adult cochlea samples which usually have higher charging even after platinum coating.

For immunogold SEM, the use of a backscatter detector allows detection of colloidal gold and thus localization of specific proteins. With IG-SEM, the protein of interest has to be on the surface—either extracellular or exposed—so the antibody-conjugated gold particles can reach the epitope at the time of labeling and are also accessible to the electron beam. Therefore IG-SEM can be an ideal tool for analyzing the composition and spatial distribution of stereocilia surface specializations. Immunogold samples can be concurrently imaged with a backscatter detector to detect gold distribution, and with a secondary electron detector for hair-cell surface topology. For immunogold labeled and 5-nm-palladium-coated samples, 10 kV with 50 μA current provides an adequate image of the stereocilia and good detection of gold beads at the surface of stereocilia (Figure 5).

One of the advantages of SEM is the depth-of-field capacity. Relatively large objects like dozens of hair cells can be imaged—all in focus—when the working distance is set accordingly. Shortening the working distance improves the resolution but limits the focal depth. For high-resolution images, a working distance of ∼6 mm is generally quite good. For low magnification images, a working distance of 6–10 mm is better. A final advantage of SEM is that it is usually non-destructive: samples can be imaged multiple times with lower or higher magnification or to re-examine them for new features. Note, though, that stereocilia bundles are often prone to charging, and high magnification imaging of a single hair bundle can cause visible damage.

#### Conventional and Immunogold Focused-Ion-Beam Scanning Electron Microscopy

The development of powerful microscopes, hybrid imaging technologies (FIB-SEM, SBF-SEM), digital image acquisition, and increased computer storage and speed have enabled 3-D reconstruction of large data sets from large volumes with high resolution (Bullen et al., 2015; Katsuno et al., 2019; Wu et al., 2019; Hadi et al., 2020; Ivanchenko et al., 2020a; Hua et al., 2021; Lu et al., 2021; Payne et al., 2021). One of these promising approaches is FIB-SEM, which generates 3-D images of large volumes with a resolution close to that of TEM. In FIB-SEM, a sample is embedded in a resin block, as for sectioning for traditional TEM, but thin sections are sequentially milled off by an ion beam. Each freshly generated block surface is imaged with an SEM. By repeating the milling and imaging hundreds or thousands of times, a serial 3-D data set is generated as a stack of consecutive images. Usually, the serial images are obtained with the SEM backscatter imaging mode and appear inverted (compared to TEM images) so that high electron-dense areas (heavy metal stained) show up as light and low electron-dense areas are dark (Figure 8). The volumetric data set is then aligned, segmented and 3-D reconstructed using Dragonfly, Amira, or other software packages (Ivanchenko et al., 2020a).

Focused-ion-beam scanning electron microscopy can produce high-resolution data sets of large volumes (Ivanchenko et al., 2020a), but the imaging sessions are time-consuming and expensive. Finding an appropriate area of interest is necessary for obtaining reliable serial images within a reasonable time. A specific site usually is pre-selected with the overview image taken from flat block surfaces of the exposed tissue using a backscatter detector. Pre-selection requires deep knowledge of cochlear morphology. As we previously described (Ivanchenko et al., 2020a), appropriate zoom and milling steps are chosen and the area is imaged at high resolution, for instance with the Auto Slice and View G3 operating software associated with the FEI Helios 660 FIB-SEM.

In FIB-SEM, high resolution is generally achieved at the cost of volume, whereas high volumes are achieved at the cost of resolution. The optimal parameters vary widely with the scientific question and compromise often is necessary. In modern FIB-SEM microscopes, such as the FEI Helios 660, overnight imaging can generate volumes of up to 20 μm × 20 μm × 10 μm, with a milling step (virtual slice thickness) of 5–20 nm. Resolution near ∼2–3 nm can be achieved. Another benefit of FIB-SEM—besides the improvement of X-Y-Z resolution—is the ability to image a small volume of the sample without destroying the rest of the block surface, which allows subsequent milling of other regions of interest from the same sample (Peddie and Collinson, 2014).

What resolution do we need for hair-cell imaging? The resolution obtainable in TEM is 0.2–0.5 nm, and similar resolution is also achievable in SEMs where the size of the electron probe ultimately limits the resolution to 0.5–2 nm. The FIB-SEM provides X-Y resolution near ∼2–3 nm and Z-resolution is the user-defined milling thickness of 5–20 nm. For stereocilia as small as 100 nm in diameter, the pixel size in any dimension should ideally be less than 20 nm. However, much higher X-Y resolutions of 1–5 nm are required to visualize subcellular structures such as tip links, actin filaments, and organelles, or the gold beads on the immunolabeled samples. There is also no single opinion about the Z-step. From our experience, 10–20 nm is good enough to obtain reliable data sets for 3-D reconstruction of hair cell bundles. It is important to note that the electron beam images the freshly exposed surface, but it also penetrates into the resin block, so some of the high-contrast information collected from the scan belongs to the tissue positioned slightly below the surface. For example, a 12-nm gold bead yielding high-contrast backscatter electrons is visible 1–2 slices before it is exposed at the surface of the resin block, at which point it will display its maximum signal intensity.

Although conventional FIB-SEM does not provide information about protein composition or localization, FIB-SEM on immunogold-labeled samples can be used for high-resolution volumetric protein mapping. With the 3-D data set, one can computationally segment subcellular or suborganellular regions with high precision and evaluate spatial distribution of gold beads within that region, enabling a quantitative composition–location–ultrastructure correlation (Ivanchenko et al., 2020a). Labeling of extracellular proteins of interest is compatible with preservation of ultrastructure, and while it is possible to perform immunogold-FIB-SEM in samples with intracellularly labeled proteins, use of permeabilization agents such as Triton X-100 or saponin is unavoidable, resulting in widespread ultrastructural damage (Bozzolla and Russell, 1998). Some other limitations apply: during the “slice and view” process the cell can’t be reexamined because of the destructive nature of the technique; there is no way to increase the contrast with post-staining; the tissue needs to be heavily stained before embedding in resin; and the FIB-SEM method requires expensive specialized equipment and highly specialized user training.

Despite the resolution and versatility of all these EM techniques, they still require a deep understanding of methods used and design of proper controls. Postprocessing and analysis of EM data should be carried out by experts with experience in interpreting specific features in the grayscale EM world. The manual segmentation and analysis of large datasets is time-consuming. Speed and insight may come from new machine-learning based tools that can automate segmentation, reconstruction, and data analysis, which in turn would bring tremendous progress to the field. Some progress has been made in the development of machine learning algorithms (Hagita et al., 2018), but they have been limited to defined structures such as mitochondria (Dietlmeier et al., 2013), synapses (Lin et al., 2020), and muscle (Caffrey et al., 2019). Despite a high level of collaboration in the field, online sharing of methods and data analysis code, and terabyte-sized data sets, many automated methods are not accessible to beginners or non-experts and are limited to the specific applications for which they were developed. They may need to be optimized or trained again for other applications. Furthermore, development of machine learning algorithms is also a time-consuming and extensive process that requires lots of manually generated training data and will be carried out by experts in the field. Still, we can expect more powerful and greater accessibility of sophisticated analysis algorithms as data science is integrated with these advances in imaging physics.

### Conclusion

In summary, a number of EM techniques can be chosen to generate structural data at a range of resolutions matched to the research question and capabilities. Although guidelines like the ones described above are available, the best approach preferably should be selected with the help of an expert in the field. A large variety of strategies and EM tools is now widely accessible and is no longer restricted to a few electron microscopy labs providing versatility of obtained data.

## Data Availability Statement

The original contributions presented in the study are included in the article/Supplementary Material, further inquiries can be directed to the corresponding author.

## Ethics Statement

The animal study was reviewed and approved by the Animal Care Committee of Harvard Medical School.

## Author Contributions

MI carried out conventional and immunogold TEM, SEM, and FIB-SEM microscopy, analyzed the data, created figures, and wrote the manuscript. AI carried out anti-STRC SEM and FIB-SEM microscopy, analyzed the data, oversaw the project, and helped to write the manuscript. DC analyzed the data, oversaw the project, and helped to write the manuscript. All authors contributed to the article and approved the submitted version.

## Conflict of Interest

The authors declare that the research was conducted in the absence of any commercial or financial relationships that could be construed as a potential conflict of interest.

## Publisher’s Note

All claims expressed in this article are solely those of the authors and do not necessarily represent those of their affiliated organizations, or those of the publisher, the editors and the reviewers. Any product that may be evaluated in this article, or claim that may be made by its manufacturer, is not guaranteed or endorsed by the publisher.

## Footnotes

1
https://hereditaryhearingloss.org

## Supplementary Material

Supplementary Movie 1 3-D localization of PCDH15 in a P1 IHC using immunogold FIB-SEM. A 3-D reconstruction of an OHC stereocilia bundle from 426 serial FIB-SEM cross sections, at a 10 nm milling step. Tissue was labeled with anti-PCDH15 and a gold-conjugated secondary antibody (12 nm). Yellow, gold beads; blue, stereocilia; gray, cell body.
